# Increased specificity of *Fasciola hepatica* excretory-secretory antigens combining negative selection on hydroxyapatite and salt precipitation

**DOI:** 10.1038/s41598-024-54290-8

**Published:** 2024-02-16

**Authors:** Florencio M. Ubeira, Marta González-Warleta, Victoria Martínez-Sernández, José Antonio Castro-Hermida, Esperanza Paniagua, Fernanda Romarís, Mercedes Mezo

**Affiliations:** 1 Laboratorio de Parasitología, Facultad de Farmacia, 15782 Santiago de Compostela, Spain; 2 Instituto de Investigación en Análisis Químicos y Biológicos (IAQBUS), Universidad de Santiago de Compostela, 15705 Santiago de Compostela, Spain; 3 Laboratorio de Parasitología, Centro de Investigaciones Agrarias de Mabegondo, AGACAL, 15318 Abegondo (A Coruña), Spain; 4 Servicio de Dermatología Médico-Quirúrgica y Venereología, Complejo Hospitalario Universitario de Pontevedra (CHUP), 36071 Pontevedra, Spain

**Keywords:** Biochemistry, Biological techniques

## Abstract

A single and rapid method to obtain an antigenic fraction of excretory-secretory antigens (ESAs) from *Fasciola hepatica* suitable for serodiagnosis of fascioliasis is reported. The procedure consists in the negative selection of *F. hepatica* ESAs by hydroxyapatite (HA) chromatography (HAC; fraction HAC-NR) followed by antigen precipitation with 50% ammonium sulphate (AS) and subsequent recovery by means of a Millex-GV or equivalent filter (Fi-SOLE fraction). Tested in indirect ELISA, the Fi-SOLE antigens detected natural infections by *F. hepatica* with 100% sensitivity and 98.9% specificity in sheep, and 97.7% sensitivity and 97.7% specificity in cattle, as determined by ROC analysis. The SDS-PAGE and proteomic nano-UHPLC-Tims-QTOF MS/MS analysis of fractions showed that the relative abundance of L-cathepsins and fragments thereof was 57% in fraction HAC-NR and 93.8% in fraction Fi-SOLE. The second most abundant proteins in fraction HAC-NR were fatty-acid binding proteins (11.9%). In contrast, free heme, and heme:MF6p/FhHDM-1 complexes remained strongly bond to the HA particles during HAC. Interestingly, phosphorylcholine (PC)-bearing antigens, which are a frequent source of cross-reactivity, were detected with an anti-PC mAb (BH8) in ESAs and fraction HAC-NR but were almost absent in fraction Fi-SOLE.

Fascioliasis (= fasciolosis) is a broadly extended parasitic disease caused by digenean trematodes of the genus *Fasciola*. This genus comprises three species: *F. hepatica* and *F. gigantica*, both of which can infect humans and livestock, although with different geographical distribution^1,2^, and *F. nyanzae* reported only in hippos^3^. There is a fourth species, *F. jacksoni*, which infects elephants, but some morphological and molecular studies suggest it is better placed in the *Fascioloides* genus^4^.

The in vitro diagnosis of human and animal infections by *F. hepatica* and *F. gigantica* can be done by microscopical examination of faecal samples to reveal the presence of ova, by detecting coproantigens in stools, by detecting anti-*Fasciola* antibodies in serum, plasma and other biological fluids, and by molecular techniques^5^. Regarding antibody detection, native L-cathepsins, which are abundantly secreted by the adult parasites when cultured in vitro, are among the most convenient target antigens in terms of sensitivity and specificity^6–8^. However, purification of L-cathepsins from whole excretory/secretory antigens (ESAs) is expensive and time-consuming, as these antigens are complex mixtures and are frequently contaminated with other secreted antigens (e.g., Fh-HDM1/MF6p^9,10^), non-secreted antigens (e.g., tegument glycoproteins^11^), and host proteins^12,13^. Consequently, some commercial and non-commercial (in house) immunological in vitro diagnostic devices which use *Fasciola* native antigens frequently lack enough sensitivity and/or specificity, particularly when testing sera from animal species in field studies. A way to overcome this problem is the use of specific anti-cathepsin monoclonal antibodies (mAbs) for their purification either using affinity columns or for direct antigen capture into the wells of ELISA plates^7,14^. However, when specific mAbs are not available, or when the use of high-quality commercial devices is not affordable, the use of purified native L-cathepsins from *Fasciola* ESAs is still recommendable. In addition, the development of single methods for purification/enrichment of L-cathepsins from *Fasciola* ESAs is also of interest for other applications, including antigen purification for production of polyclonal antibodies, for research studies on native cysteine-proteases, and, possibly, for the development of new experimental vaccines.

In the past years, purification of *Fasciola* L-cathepsins from ESAs was achieved by combining several chromatographic strategies as, for example, Sephacryl S-200 size-exclusion chromatography (SEC) followed by QAE-Sephadex chromatography^15^, a two-step alcoholic fractionation followed by ion-exchange chromatography^16^ and a one-step ethanol fractionation followed by chromatography on carboxymethyl sephadex C-50 columns^17^. A L-cathepsin rich fraction from *F. hepatica* was also previously obtained by our group by SEC on a Superdex 75 HR 10/300 column (peak IV), which was useful for serodiagnosis of *F. hepatica* infections in sheep^18^. However, none of these methods are suitable in terms of simplicity, costs and yield when semi-preparative purifications are required. In the present study we observed that *F. hepatica* ESAs can be sieved by hydroxyapatite chromatography (HAC) followed by ammonium sulphate (AS) salt precipitation of the proteins present in the non-retained fraction to obtain an L-cathepsin rich fraction useful as target antigen in ELISA and for other applications. The nature of the proteins obtained throughout the enrichment procedure was determined by shotgun nHPLC-MS/MS analysis/sequencing.

## Methods

### Ethic declarations

This study was carried out in strict accordance with the guidelines of European Directive 2010/63/EU and Spanish Law RD 53/2013 on the Care and Use of Laboratory Animals. The protocol was approved by the Ethics Committee of the Consellería do Medio Rural of the Xunta de Galicia (Spain). The authors complied with the ARRIVE guidelines (https://arriveguidelines.org).

### Obtaining excretory-secretory *Fasciola* antigens (ESAs)

*F. hepatica* ESAs were obtained as previously described^18^. Briefly, live adult flukes collected from bile ducts of naturally infected sheep were washed, first in sterile saline solution containing glucose (2 g/L) at 38°C and then in RPMI 1640 cell culture medium (Merck Life Science SLU, Madrid, Spain) supplemented with 20 mM HEPES, 0.3 g/L L-glutamine, 2 g/L sodium bicarbonate and antibiotics (100 IU/mL penicillin and 100 µg/mL streptomycin) at 38 °C. Flukes were then transferred to 75  cm^2^ tissue culture flasks and maintained in culture medium (3 mL/fluke) at 38 °C under 5% CO_2_ in air. After incubation for 24 h, the medium containing ESAs was removed and centrifuged at 10,000 *g* for 30 min at 4°C in the presence of protease inhibitors (SigmaFast Protease Inhibitor Tablets; Merck Life Science SLU). The supernatant was passed through a 0.45 µm pore filter and concentrated in an Amicon 8050 ultrafiltration cell (Merck Life Science SLU) equipped with YM10 membrane (10 kDa cut-off), dialyzed against phosphate-buffered saline (PBS; 10 mM sodium phosphate, 150 mM NaCl, pH = 7.2), sterilized by filtration (0.22 µm pore size), and stored at -80°C until required.

### Serum samples

#### Sheep sera

Serum samples (N = 90) were collected from adult grazing sheep belonging to 9 commercial flocks considered as ‘*Fasciola*-free’ on the basis of two criteria: (i) liver fluke infection was not detected, either by routine coprology or necropsies, in the last 5 years; (ii) each sheep from these flocks was negative for anti-*Fasciola* antibodies by the MM3-SERO ELISA test^14^. Serum samples (N = 40) from *Fasciola*-infected sheep were collected from 10 naturally infected flocks. All sampled sheep were confirmed to shed both *Fasciola* eggs (detected by coproscopy) and coproantigens (detected by the enhanced MM3-COPRO ELISA test, according to Mezo et al*.*^19^).

#### Cattle sera

Serum samples (N = 44) from grazing adult cattle were obtained from 3 *Fasciola*-free herds. Classification of a herd as ‘*Fasciola*-free’ was based on the criteria described above for sheep flocks. Serum samples (N = 44) from infected cattle came from: (i) 27 animals with natural infection confirmed by detection of both *Fasciola* eggs and coproantigens in the faeces; (ii) 17 animals sacrificed at slaughterhouse in which the presence of adult flukes was confirmed by ocular inspection of the liver. All *Fasciola*-free sheep and cattle were demonstrated to be infected by several of the helminths or protozoa included in Table 1. Analyses were performed by using traditional coprological techniques^19^.

**Table 1:** Helminths and protozoa identified in *Fasciola*-negative sheep (A) and cattle (B).

A	
Nematodes	*Cystocaulus ocreatus* *Muellerius capillaris* *Protostrongylus* spp. *Nematodirus* spp. *Trichuris* spp. Other strongylids, unidentified genus
Trematodes	Paramphistomidae, likely *Calicophoron* spp. *Dicrocoelium* spp.
Cestodes	*Moniezia* spp.
Protozoa	*Eimeria* spp. *Balantidium*-like ciliates

B	
Nematodes	*Nematodirus* spp. *Trichuris* spp. Other strongylids, unidentified genus
Trematodes	Paramphistomidae, likely *Calicophoron* spp. *Dicrocoelium* spp.
Cestodes	*Moniezia* spp.
Protozoa	*Eimeria* spp. *Balantidium*-like ciliates

### Total protein and sugar measurements

The concentration of protein in whole and fractionated *F. hepatica* ESAs was measured using the Pierce BCA Protein Assay Kit (Thermo Fisher Scientific, Barcelona, Spain). In addition, OD_280_/2.4 values were also provided at the end of the purification process for comparison. The 2.4 coefficient was calculated from the quotient between the extinction coefficient (59,300 M^−1^ cm^−1^) and the MW (24.51 kDa) of the secreted portion of a recombinant secreted L1-cathepsin^6^. When required, total sugar content of samples was determined by a modification of the phenol–sulphuric method^20^ consisting in premixing 500 μL of 5% phenol with 500 μL of each sample to which 2.5 mL of concentrated sulphuric acid is added. After 10 min at room temperature (RT), the tubes were cooled with tap water for 15 min, vortexed, and the absorbance measured at 490 nm. Known concentrations of glucose were used to construct the calibration curve.

#### Hydroxyapatite fractionation of ESAs

A chromatographic column (1.5 ID × 10 cm; CrystalCruz™ Chromatography Columns, Santa Cruz Biotechnology, Inc., Heidelberg, Germany) was filled with hydroxyapatite (HA) powder resuspended in PBS (Bio-Gel HTP Hydroxyapatite; Bio-Rad, Madrid, Spain) following the instructions provided by the manufacturer. For this, 5 g of powdered HA were weighed and added to a 50 mL plastic falcon tube containing 30 mL of PBS, pH 7.2. The tube was then capped and gently shaken by inversion and placed in a vertical position for 10 min to allow the suspension to settle. Fines in the cloudy top level were then aspirated and the settled particles were resuspended with PBS and allowed to settle again. Finally, the supernatant was aspirated, the settled particles resuspended in PBS, and the particle suspension used to pack the column. Once filled with the HA particles, the column was connected to a Cytiva Acta Start protein purification system (VWR International Eurolab, Barcelona, Spain) and equilibrated with PBS for 30 min with a flow rate of 2 mL/min (manual run). Then, the input flow was closed, and the system paused after the remaining buffer on the top of the resin entered into the column bed.

**Figure 1:**
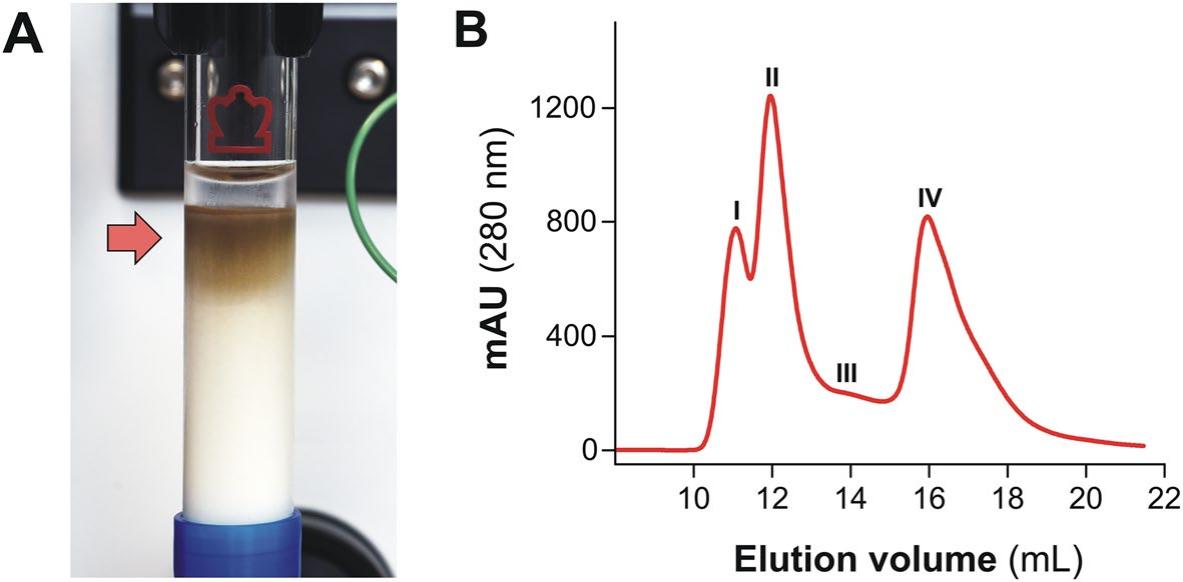
(**A**) Photography of a HA column showing the brownish product retained at the top of the column (arrow) during the chromatographic separation of *F. hepatica* ESAs. (**B**) Elution profile of *F. hepatica* ESAs obtained by size exclusion chromatography (Superdex 75 HR 10/30, ÄCTA LC) monitored at 280 nm. The fraction corresponding to Peak IV was used for comparative studies with fractions obtained by HAC.

For sample preparation, 8 mL of *Fasciola* ESAs were centrifuged at 20,000 *g *for 15 min at 4 °C in a 5425R microcentrifuge (Eppendorf SE, Hamburg, Germany) equipped with a FA-10 × 5 rotor. Subsequently, the supernatant was collected, and a 7.5 mL sample containing 13.8 mg of protein, as measured by BCA, was collected and pipetted carefully on the top of the column bed and chromatographed while monitoring the protein concentration at 280 nm. To minimize dilution of the protein during the chromatographic procedure, the sample was allowed to enter the chromatographic matrix before PBS was added again to the top of the HA column (see Fig. 1A). Two consecutive fractions were collected: (i) a non-retained (NR) fraction which was collected in the flow-through (HAC-NR), and (ii) a retained (RET) fraction eluted with 0.5 M sodium phosphate buffer, pH 7.2 (HAC-RET). After elution, the collected samples were provisionally stored at 4 °C, and the column washed with 10 volumes of 0.3 N NaOH and stored in the same solution at 4 °C. Then, the protein concentration of each fraction was measured, and the samples were stored at −20 °C.

### Purification of *Fasciola* antigens by SEC

Antigens corresponding to Peaks I, II and IV^18^ were purified from whole *F. hepatica* ESAs by size exclusion chromatography (SEC) using a high-performance liquid chromatography system (ÄKTA Basic 10, Amersham Biosciences Europe GmbH, Barcelona, Spain) with a Superdex 75 HR 10/300 column (Amersham Biosciences; see Fig. 1B). The column was calibrated with a mixture of known-molecular weight proteins (Gel Filtration LMW Calibration Kit, Amersham Biosciences). Briefly, samples of 0.5 mL (protein concentration 2 mg/mL) were loaded onto the column equilibrated with PBS and then eluted at a flow rate of 0.5 mL/min. The effluent was monitored for protein concentration at 280 nm and peak fractions were collected. The fractions in the fourth peak (Peak IV), which eluted between chymotrypsinogen A (25 kDa) and ribonuclease A (13 kDa), were pooled, concentrated by ultrafiltration using Centricon YM-10 centrifugal filter devices (Merck Life Science SLU) and stored at -20°C until used.

### Ammonium sulphate salt precipitation of the HAC-NR protein fraction

**Figure 2:**
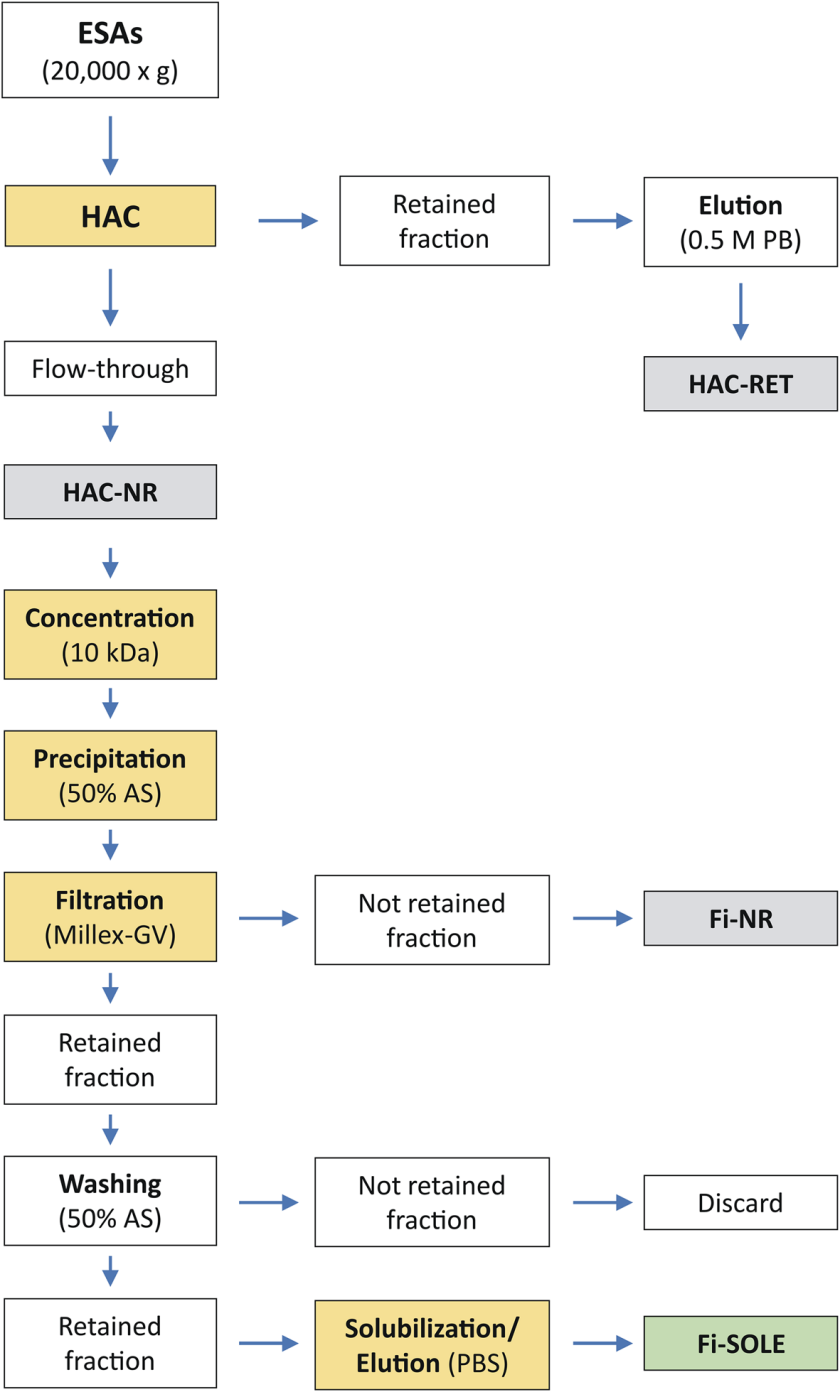
Flow chart of protein purification. The procedure includes five main steps: (**a**) isolation and collection of the flow-through biomolecules by HAC (fraction HAC-NR), (**b**) physical concentration of the HAC-NR fraction using a 10 kDa ultrafiltration membrane, (**c**) precipitation of the biomolecules of interest with saturated ammonium sulphate until 50% final concentration, (**d**) retention of the precipitated biomolecules on a 0.22 µm filter membrane (Millex-GV), and (**e**) recovery of the biomolecules of interest by solubilization/elution of the material retained by the filter (fraction Fi-SOLE).

The HAC-NR fraction was concentrated to 3 mL volume using 10K EMD Millipore Amicon™ Ultra-15 units (Merck Life Science SLU). Then, the sample was transferred to a 10-mL glass vessel and placed on ice, on a magnetic stirrer. While stirring, the same volume of saturated solution of AS was added drop by drop with the aid of a micropipette and the solution maintained in agitation for 30 min. Afterwards, the fluid containing the protein precipitates was collected with the aid of a 10-mL disposable syringe and filtered through a low-protein binding, 0.22 µm, PVDF membrane disposable filter (Millex-GV®, 25 mm diameter; Millipore-Merck) and the filtrate (first filtrate) collected for subsequent dialysis against PBS (fraction Fi-NR; Fig. 2). Thereafter, the proteins retained in the filter were washed with 2 mL of 50% AS and the filtrate discarded (second filtrate). Immediately, the walls of the vessel used for protein precipitation were washed with 2 mL PBS to dissolve any remaining protein, the fluid collected with the syringe, and passed slowly through the Millex-GW filter to dissolve the retained proteins and the filtrate collected. Washing cycles with 2 mL of fresh PBS were repeated until the concentration of protein in the filtrate was negligible as determined by OD_280_ (two times in our case). Finally, the collected filtrates were combined into a single fraction (Fi-SOLE; Fig. 2), the protein concentration measured, and stored in aliquots at -20 °C for further uses. The filtration procedure was preferable to centrifugation to collect the protein since, in our experimental conditions, precipitates of L-cathepsin have less density than the 50% AS solution and are retrieved as floating pellets even when centrifuged at medium/high speed (e.g., 20,000 × *g*). A flow chart of all steps followed for protein purification combining HAC and AS precipitation is showed in Fig. 2.

### Spectrophotometric analysis of HA and Superdex 75 HR chromatographed fractions

A major contaminant observed during the in vitro culture of adult flukes is the presence of large aggregates of the heme-binding protein MF6p/FhHDM1 basic protein^9,10^, as well as free hemin produced as the result of enzymatic digestion of haemoglobin from host red blood cells by action of *Fasciola* proteases, mainly L-cathepsins^21^. To assess the presence of heme derivatives in the different protein fractions, whole ESAs, samples corresponding to peaks I, II and IV obtained by SEC (Superdex 75 HR 10/300; see Fig. 1B), and two of the samples obtained after HAC (HAC-NR and HAC-RET; see Fig. 2), were diluted appropriately in PBS and scanned for UV/VIS absorption in the range of 250–650 nm using a Multiskan GO spectrophotometer (Thermo Fisher Scientific).

### SDS-PAGE analysis

Protein aliquots from Peak IV, HAC-NR and Fi-SOLE were analysed by SDS-PAGE under denaturing conditions and subsequently by LC–MS/MS. The samples diluted at the desired protein concentration with dH_2_O, were prepared by mixing them 1:1 with 2X sample loading buffer (composed by 2.5 volumes of 4X LDS sample buffer (GenScript Biotech, Leiden, Netherlands) + 1 volume of 1M DTT + 1.5 volumes of dH_2_O) and then boiled for 10 min. For SDS-PAGE analysis, we used SurePAGE, Bis–Tris, precast mini polyacrylamide gels (8–16% gradient, 10 cm × 8 cm, 12 wells, GenScript Biotech), used according to the manufacturer instructions. The gel tank was a Hoefer Mighty Small (SE 250) and the running buffer was Tris-MOPS-SDS, also from GenScript Biotech. Pre-stained MW markers were from Thermo Fisher Scientific (PageRuler™ Plus, 10–250 kDa). After electrophoresis, the gel was removed from the cassette, rinsed with dH_2_O, and then stained overnight with BlueSafe™ (Nzytech, Lisboa, Portugal) under orbital shaker (50 rpm). Next, the gel was washed for 2 min with ethanol to eliminate the colorant excess and then with several changes of dH_2_O. Finally, the gel was photographed, and the bands of interest were excised for LC–MS/MS analysis (see below).

### Mass spectrometric analysis

The identification of proteins in bands isolated by 1D SDS-PAGE was done at the Mass Spectrometry and Proteomic Unit (Universidad de Santiago de Compostela, Spain) using nanoUHPLC-Tims-QTOF technology. Specifically, we used a TimsTof Pro Mass Spectrometer (Bruker Daltonik GmbH, Fällanden, Germany) equipped with a nanoELUTE chromatograph (Bruker Daltonik GmbH) and a nanoESI (CaptiveSpray) ion source. For sample preparation, the bands excised from the SDS-PAGE gel were distained with ethanol, digested with trypsin, reduced/alkylated with dithiothreitol-iodoacetamide, desalted, and concentrated using pipette tips (ZipTip with 0.6 µL C18 resin; Millipore-Merck) and finally dissolved in 0.1% formic acid before analysis. For peptide separation we used a ReproSil C18, 150 × 0.075 mm, 1.9 µm, 120Å (Bruker FIFTEEN) column, 2 µL samples, temp 50°C, and a mobile phase gradient composed by H_2_O/0.1% formic acid and acetonitrile/0.1% formic acid. Automated software for data analysis was done by Compass HyStar 5.1 (Bruker Daltonik GmbH) and oTOF Control 6.2 (Bruker Daltonik GmbH) for data acquisition. The acquisition parameters were as follows: method (DDA-PASEF); ionization mode (nanoESI positive); scan mode (PASEF-MSMS); fragmentation mode (CID); m/z acquisition range (100–1700). Software for data processing were DataAnalysis 5.3 (Bruker Daltonik GmbH) and PEAKS Studio 10.6 (Bioinformatics Solutions). Searches were restricted to *Fasciola hepatica* taxonomy (FasciolaHepatica_NCBI_010422) and the Database for contaminants was Contaminants_MaxQuant1.6.17.0. The searching parameter were as follows: precursor mass error tolerance: 15.0 ppm; fragmentation error mass tolerance: 0.02 Da; enzyme: trypsin; digestion mode: semi-specific; fixed modifications (carbamidomethylation): 57.02; variable modifications: acetylation (protein N-term): 42.01)/ deamidation (NQ): 0.98/ oxidation (M): 15.99/ dehydration (-18.01); maximal number of post-translational modification per peptide: 3.

### ELISA methods

#### Quantification of L-cathepsins in chromatographic fractions of *Fasciola* ESAs obtained by HAC

To quantify L-cathepsins in the peaks obtained after fractionation of *Fasciola* ESAs by HAC (HAC-NR and HAC-RET, see above), we used the MM3-COPRO ELISA previously reported^22^. Specifically, the proteins in each peak were first adjusted at a starting concentration of 40 ng/mL in CoproGuard (Inmunogal, Santiago de Compostela, Spain), then diluted serially (1/2 dilutions in CoproGuard), and the amount of L-cathepsins in each sample estimated by MM3-COPRO ELISA. In parallel, a reference curve was done using whole *Fasciola* ESAs with known protein concentration as control. A cut-off value of OD = 0.02 previously determined for this assay was used to calculate the dilution limit of the assay at which L-cathepsin signal still is detected.

#### Anti-*F. hepatica* antibody detection

An indirect ELISA (iELISA) was developed for determinations of anti-*Fasciola* antibodies in sera from infected and non-infected animals (sheep and cattle). Four *Fasciola* target antigens, ESAs, Peak IV, HAC-NR and Fi-SOLE were evaluated. Firstly, the optimal dilution for each antigen to be tested was calculated by coupling an ELISA plate (12 × F8 strips; Greiner Bio-One, Madrid, Spain) with one half serial dilutions of each antigen in PBS and further analysis of their reactivity using biotinylated MM3 mAb as primary antibody, streptavidin-polyHRP (Pierce; Thermo Fisher Scientific) as amplification reagent, and TMB-ONE (Kementec, Taastrup, Denmark) as enzyme substrate. Protein concentrations of 1 µg/mL for *Fasciola* ESAs and of 0.5 µg/mL for the remaining antigens (HAC-NR, Fi-SOLE and Peak IV) were selected.

After choosing the antigen concentrations, the ELISA plates for antibody detection (Greiner Bio-one) were coated overnight with the corresponding antigen diluted in PBS at the above concentrations, then washed 3X with PBS and blocked with 1.5% sodium caseinate for 2h at RT. Next, the plates were aspirated and incubated with the corresponding sera from infected and non-infected animals. For detection of antibodies in sheep sera, the samples were diluted 1:400 when plates were coated with ESAs, and 1:200 for plates coated with the remaining antigens. The iELISA included the following steps: (1) addition (100 µL/well) of each diluted sample in duplicate; (2) addition (100 µL/well) of peroxidase-conjugate anti-sheep IgG mAb (dilution 1:20,000; Merck Life Science SLU); (3) addition (100 µL/well) of the TMB substrate (TMB ONE™, Kementec; incubation time 20 min in the dark) and (4) addition (100 µL/well) of 0.2 M H_2_SO_4_. Dilutions of samples and conjugate were made in PBS containing 0.2% Tween 20 (PBS-T) and 1% dry skimmed milk. Incubations were carried out at 37 °C for 2 h (serum samples) or for 1 h (conjugate). After incubations, the plates were washed 6 times with PBS-T using an automated 96-channel microplate washer (Agilent BioTek 405 TS Microplate Washer; BioTek instruments, Winooski, VT, USA). The OD was measured at 450 nm with a spectrophotometer (Tecan Spectra Rainbow A-5082; Tecan Ibérica Instrumentación SL, Barcelona, Spain). Negative and positive control samples were included in every plate. The ELISA IgG values were expressed as Log_10_ OD response measured at 450 nm.

Regarding cattle sera, the iELISAs were performed as described above, with the following modifications: (i) sera were diluted 1:100 for iELISAs with Peak IV, HAC-NR and Fi-SOLE as target antigens; (ii) two conjugates were used: first, a monoclonal anti-bovine IgG (Ingenasa, Madrid, Spain) labelled with FITC, and then, a peroxidase-conjugated rabbit anti-FITC IgG (Abcam, Cambridge, UK). The dilution used for both conjugates was 1:5000.

#### Determination of PC-bearing molecules in fractionated *Fasciola* ESAs

To determine the presence of phosphorylcholine (PC)-containing molecules in fractions of *Fasciola* ESAs obtained by HAC and in peak IV (see above), we designed an iELISA for which the different antigens were coated to ELISA plates (12 × F8 strips; Greiner Bio-One). The concentrations of the antigens were adjusted at 10 µg/mL in PBS measuring the corresponding absorbances at 280 nm in comparison with the PC-ovalbumin (PC-OVA) used as a positive control. The coated plates were incubated overnight at 4°C, washed 3 times with PBS and then blocked with 1.5% sodium caseinate at RT for 1h. Next, the wells of the ELISA plates were incubated for 30 min with serial one-half dilutions of an anti-PC IgM mAb (BH8; see Romarís et al*.*^23^) diluted in PBS containing 0.05% Tween-20 and 1% BSA (PBS-T-B), starting at 1/5000 dilution. After 6 washing cycles with PBS-T, the plates were incubated with a peroxidase-conjugated rabbit anti-mouse antibody (Dako Diagnósticos SA, Barcelona, Spain) diluted 1/2000 in PBS-T-B for 30 min at RT with orbital shaking as above. Then, the plates were washed again with PBS-T (6 cycles) and incubated with 100 µL/well of TMB-ONE for 10 min at RT in the dark. Next, the reaction was stopped by adding 100 µL/well of a stop solution (0.2 M H_2_SO_4_). Finally, the absorbance was read at 450 nm with a Thermo Scientific™ Multiskan™ GO Microplate Spectrophotometer (Thermo Fisher Scientific).

### Immunohistochemistry (IHQ)

Immunocytolocalization of the PC epitope on *Fasciola* adult worms recognized by mAb BH8 was done as previously reported^9,24^. Briefly, fresh worms obtained in the slaughterhouse were fixed in 10% buffered formalin for 24 h at RT, washed twice with PBS to remove excess fixative, dehydrated through graded series of alcohol, and cleared with xylene. Next, the flukes were cut into small transversal pieces, embedded in Histosec (Merck Life Science SLU), sliced into 5-µm-thick sections using a Jung Supercut 2065 microtome (Leica, Nussloch, Germany), and mounted on slides. The sections were dewaxed and rehydrated through xylene and a decreasing ethanol gradient and finally rinsed with distilled water. Afterwards, the sections were blocked with Tris-buffered saline (TBS) containing 0.05% Tween 20 and 1% dry skimmed milk (TBS-T-SM) at RT for 2 h and then incubated 1 h at RT with mAb BH8 (1:1000). After washing the slides three times with TBS-T, the sections were incubated with peroxidase-labeled rabbit anti-mouse Ig antibody (dilution 1:800 Dako Diagnósticos SA) diluted in TBS-T-SM for 1 h at RT. The sections were then washed again with TBS-T and incubated for 5–10 min with 0.5 mg/mL 4-chloro-1-naphthol (Merck Life Science SLU) containing 0.005% H_2_O_2_. Thereafter, the slides were washed with TBS, mounted with glass coverslips in PBS-glycerol (1:1) and examined and photographed under an Olympus Provis AX-70 microscope equipped with a DP 70 digital camera.

### Statistical analysis

Diagnostic performance of each antigen was assessed by using receiver operating characteristic (ROC) curves calculated with Medcalc statistical software v. 20 (Medcalc Software Ltd, Ostend, Belgium). The area under the ROC curve (AUC) was calculated and then compared with the value of non-discrimination (AUC = 0.5) using the nonparametric method of Delong et al*.*^25^. The optimal cut-off values, i.e. those maximizing sensitivity and specificity, were estimated by using the Youden index. Statistical significance level was set at *P* < 0.05.

## Results and discussion

### Performance and characteristics of *Fasciola* ESAs fractionated by HAC

The *Fasciola* ESAs are complex mixtures of antigens originated from adult flukes when maintained in vitro in a culture medium for a limited period (typically, 2–24 h^12,13,18,26–29^). Most *Fasciola* ESAs are secreted by the parasites into their blind-ending gut and then regurgitated through the oral suckle during an intermittent food in/waste out cycle^30,31^. However, structural antigens released from the tegument and/or from the gut may also contribute to the diversity of antigens contained in ESAs.

When specific, the use of combined ESAs, and particularly of L-cathepsins, may be more reliable to diagnose natural *Fasciola* infections than single recombinant antigens^32^. This can be explained by at least two facts: (i) purified antigens better represent the antigenic mixture to which the host immune system is exposed during natural infections; and (ii) all components in the antigenic mixture are probably correctly folded. Purified native L-cathepsins are also advantageous over single recombinant antigens to produce polyclonal antibodies intended to develop diagnostic kits for detection of *Fasciola* antigens in biological fluids.

In an effort for searching simple analytical and/or semi-preparative methods to increase the specificity of whole *Fasciola* ESAs, in this study we investigate the usefulness of HAC to fractionate these antigens. The main strategy was to obtain a L-cathepsin rich fraction devoid of most non-specific antigens, as well as other contaminants like heme aggregates, either free or bonded to the heme-binding protein FhHDM-1/MF6p, which may be present in relevant amounts in the *Fasciola* ESAs preparations^9,10^.

HAC is broadly used in preparative biochemistry in the purification of monoclonal antibodies, proteins, and nucleic acids^33,34^. The main functional groups of HA are positively charged calcium pairs (C-sites) and negatively charged oxygen atoms associated with phosphate triplets (P-sites)^33^. HA columns are operated near to neutral pH, typically at the pH range of 6–6.8, a condition at which the column can be regarded as negatively charged and the particles are relatively stable^35^. To our knowledge, HAC was never used to purify L-cathepsins either from *Fasciola* or from other sources. However, HAC was previously used to partially purify human cathepsin H from human liver^36^. In this case, the sample to be purified was applied to the HA column in 20 mM phosphate buffer, pH 6.0, and then eluted with an increasing gradient of the same buffer (150 mM final concentration). In contrast, in our study we used HAC for the negative selection of *Fasciola* L-cathepsins contained in ESAs using PBS at pH 7.2, i.e., the same buffer previously used to dialyse *Fasciola* ESAs before storage.

As we indicated in the previous section, two main fractions (HAC-NR and HAC-RET -eluted with 0.5 M sodium phosphate-) were initially collected. In Fig. 1A it can be observed that the brown pigment (heme-containing fraction), typically present in *Fasciola* ESAs, is trapped at the top of the column (arrow). Complementarily, in Fig. 1B we show a typical chromatogram of *Fasciola* ESAs fractionated by SEC using a Superdex 75 HR column. As reported previously^18^, the heme-containing fraction is mainly concentrated in peak II while L-cathepsins eluted in peak IV. The proteins corresponding to peak IV were used for comparative studies with HAC fractions in the present study (see below).

**Figure 3:**
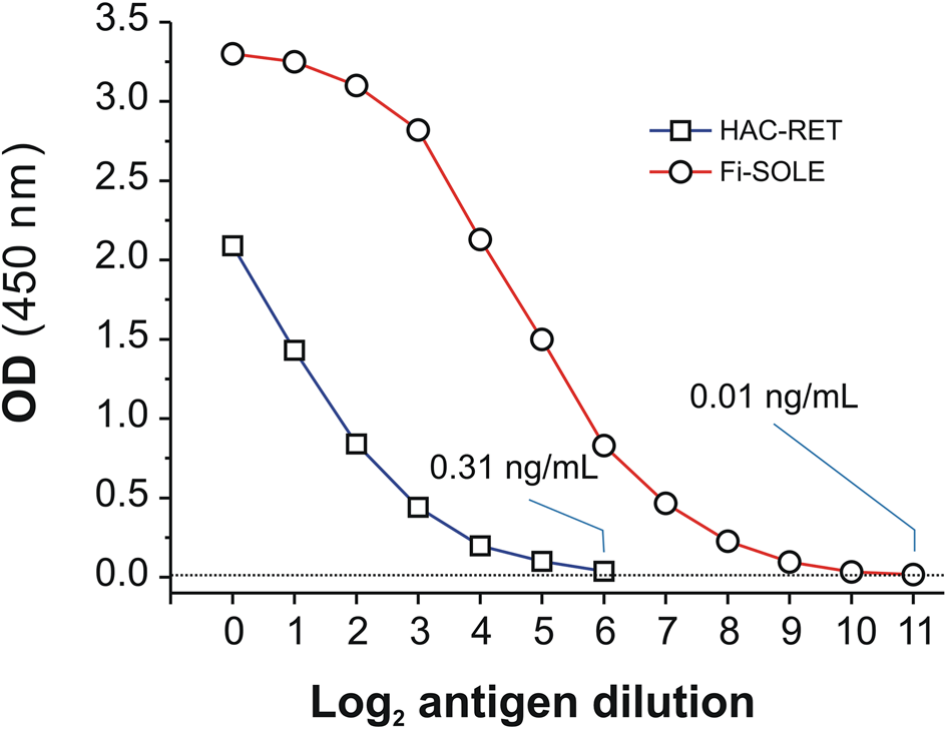
Quantification of L-cathepsins in fractions Fi-SOLE (red) and HAC-RET (blue) obtained by HAC of *F. hepatica* ESAs using the test MM3-COPRO ELISA. The graph shows the OD values obtained for each fraction measured at two-fold serial dilutions adjusted at the starting concentration of 40 ng/mL. The values in the graph indicate the concentration of total protein at the nearest dilution above the detectability of the assay (dashed line).

To evaluate the performance of HAC to isolate *Fasciola* L-cathepsins, we first measured the amount of whole protein and L-cathepsins recovered from HAC-NR and HAC-RET fractions (see Fig. 2). From the starting protein (13.8 mg), a total of 5.7 mg (41.4%) and 4.9 mg (35.5%) were recovered, respectively, from HAC-NR and HAC-RET fractions. Then, after AS precipitation of the proteins in fraction HAC-NR and subsequent filtering (fraction Fi-SOLE; see Fig. 2) a total of 4.1 mg of protein were recovered (29.7%). In contrast, the amount of protein which passed the Millex-GW filter before protein solubilization was negligible (< 5% of the incoming protein; see Fig. 2, fraction Fi-NR). It also should be noted that the percentage of protein in Fi-SOLE was only slightly lower than that obtained from peak IV after SEC of *Fasciola* ESAs using the Superdex 75 HR column (36.9% in average), also measured by the BCA method. However, when tested by the MM3-COPRO ELISA, which specifically detects L-cathepsins, the proportion of *Fasciola* L-cathepsins present in each of these fractions was very different (Fig. 3). While 96.3% of recovered L-cathepsins were present in the Fi-SOLE fraction, only 3.7% could be found in fraction HAC-RET. These percentages were extrapolated from the data of Fig. 3, considering (i) the dilution of samples required to reach the limit of detection of the assay with the test MM3-COPRO (i.e., 1/4096 for Fi-SOLE and 1/128 for HAC-RET) and (ii) that the starting dilutions for all samples were adjusted at the same concentration (40 ng/mL, measured by BCA). When the proteins from a second batch of *F. hepatica* ESAs were chromatographed using the same procedure, the percentages of L-cathepsins we obtained from fractions Fi-SOLE and HAC-RET were similar, which indicates that the proposed HAC method is reproducible. Nevertheless, as no method exists that measures with precision the amount of protein when a mixture of proteins is present in an aqueous sample, and the proportion of protein species in ESAs is variable, the calculated amount of protein in each fraction may be different. For example, the total amount of protein recovered in the final fraction Fi-SOLE dropped from 4.1 mg (measured by BCA) to 2.3 mg when the protein concentration was measured as OD_280_/2.4, i.e., considering the extinction coefficient and MW of a *F. hepatica* L1-recombinant cathepsin (see previous section). In any case, from the above results we concluded that: (i) only a minimal amount of L-cathepsins and/or their fragments present in *Fasciola* ESAs (< 5%) was retained by the HA column under the chromatographic conditions used in this study, (ii) most of the cathepsins in ESAs can be recovered in the Fi-SOLE fraction, (iii) the detection limit of the MM3-COPRO^22^ drops from 150 pg/mL, when whole ESAs are used, to about 20 pg/mL, when an enriched L-cathepsin fraction (e.g., Fi-SOLE) is used to construct the calibration curve.

**Figure 4:**
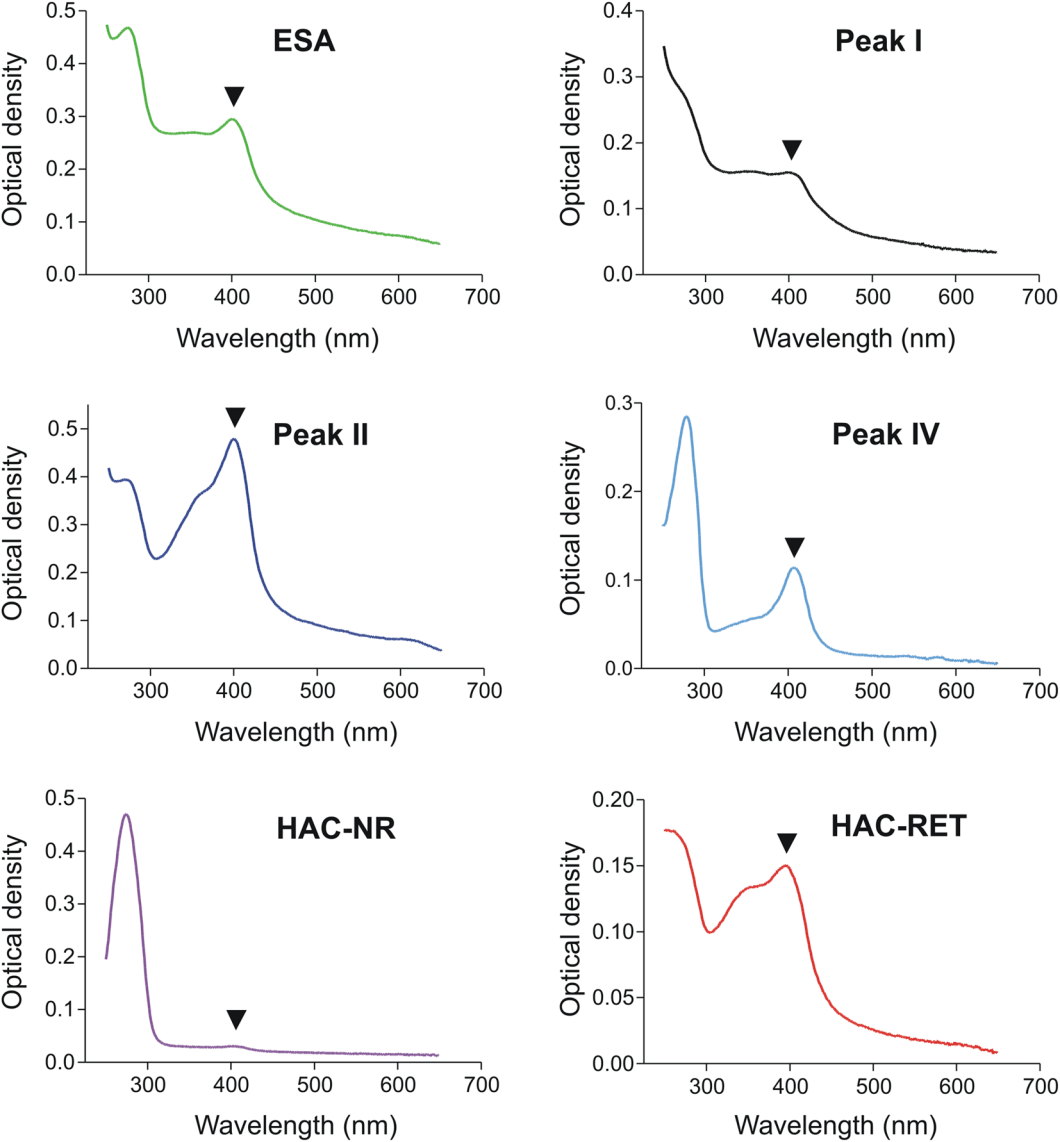
UV/VIS spectra (250–650 nm) of antigenic fractions obtained from *F. hepatica* whole ESAs, after HAC (HAC-NR and HAC-RET) and size exclusion chromatography (Peaks I, II and IV). The arrowheads show the absorbance peak (around 402 nm) corresponding to the heme group. It can be observed that only the fraction HAC-NR lacks a clear peak at 402 nm.

Regarding the ability of the HA column to retain heme and heme complexes with protein FhHDM-1/MF6p^9,37^, we carried out an UV/VIS absorption spectrum (250–650 nm scan) with each of the fractions obtained by HAC. For comparisons, we also carried out spectra of whole *Fasciola* ESAs, and the fractions corresponding to peaks I, II and IV obtained by SEC on the Superdex 75 HR column (Fig. 1B) from whole *Fasciola* ESAs. As can be seen in Fig. 4, with respect to protein content (λ_max_ 280 nm), a high proportion of heme (λ_max_ 405 nm) was present in fractions corresponding to peak II, as previously reported^18^, and in HAC-RET. However, a small, but still significant, amount of heme was also observed in peak IV. This probably corresponded to small FhHDM-1/MF6p complexes with MW near that of L-cathepsins that cannot be sieved by SEC. In contrast, the HAC-NR had negligible amount of heme, which is indicative of a very high binding affinity of heme to the HA matrix, which is according to the fact that most of the brownish pigment present in *Fasciola* ESAs remained at the top of the HA column during the entire chromatographic process (see Fig. 1A), much of which persisted bond to HA after the final wash with 1 M NaOH before storage (not showed). It is important to note that the HA column containing the Bio-Gel HTP particles can be normally regenerated using 0.5 M phosphate buffer, according to the manufacturer's instructions. However, complete removal of strong binders/insoluble substances (as in the case of heme and heme complexes present in ESAs) could not achieved even after washing with 1–2 M NaOH. So, removal of the top layer of the HA bed, or discarding the HA matrix when saturated may be necessary.

### Analysis of the antigenicity of *Fasciola* ESAs fractions isolated by HAC

Once we demonstrated that most of *Fasciola* L-cathepsins present in ESAs were collected in the flow-through (HAC-NR fraction) of HAC, we tested the sensitivity and specificity of such antigens and those remained in fraction Fi-SOLE. For comparisons, we also tested the antigens present in peak IV obtained by SEC, as well as those present in whole ESAs. So, four antigenic preparations (HAC-NR, Fi-SOLE, Peak IV and whole ESAs) were tested in parallel. The antigens in fraction HAC-RET were not tested in ELISA because they did not show enough discriminatory power in a preliminary study (not shown).

A total of 130 serum samples from sheep (40 sera from *Fasciola*-infected and 90 sera from non-infected animals) and 88 from cattle (44 sera from both infected and non-infected animals) were analysed. As no cut-off value was available for these antigens, a ROC analysis was done with each of these antigens. The results of these analyses are showed in Tables 2 (ovine) and 3 (bovine). Except for whole ESAs, the cut-off values obtained were lower for sheep than for cattle. Regarding sensitivity and specificity, whole ESAs showed, as expected, the worst results with values of sensitivity and specificity below 90% for both sheep and cattle. Moreover, the specificity of this antigen only reached a value of 56.8% for bovine serum samples (Table 3). With respect to the HAC-NR antigenic fraction, the values of sensitivity were around 95% for both animal species, but the specificity was better for sheep (98.9%) than for cattle (86.4%). These results suggested that some non-specific antigens are not retained by the HA column and elute together with *Fasciola* L-cathepsins in the HAC-NR fraction. However, after precipitation of the HAC-NR fraction with 50% AS (fraction Fi-SOLE), the sensitivity and specificity values increased, respectively, up to 100% and 98.9% for sheep, and 97.7% and 97.7% for cattle (Tables 2 and 3). These values were similar to those obtained with antigens present in peak IV (100% sensitivity and 98,9% specificity for sheep, and 97.7% sensitivity and 100% specificity for cattle), an antigenic mixture that we have already demonstrated to be sensitive and specific enough to be used as target in iELISA for the serodiagnosis of sheep infections caused by *F. hepatica*^18^.

**Table 2:** Results of ROC curve analysis in ovine negative (n = 90) and positive (n = 40) serum samples.

	Cutoff (OD)	Sensitivity (%) [95% CI]	Specificity (%) [95% CI]	AUC [95% CI]	*P*-value
ESAs	0.639	87.5 [73.2–95.8]	85.6 [76.6–92.1]	0.928 [0.869–0.966]	< 0.001
Peak IV	0.067	100 [91.2–100]	98.9 [94–100]	1 [0.971–1]	< 0.001
HAC-NR	0.178	95 [83.1–99.4]	97.8 [92.2–99.7]	0.99 [0.954–0.999]	< 0.001
Fi-SOLE	0.1	100 [91.2–100]	98.9 [94–100]	1 [0.971–1]	< 0.001

**Table 3:** Results of ROC curve analysis in bovine negative (n = 44) and positive (n = 44) serum samples.

	Cutoff (OD)	Sensitivity (%) [95% CI]	Specificity (%) [95% CI]	AUC [95% CI]	*P*-value
ESAs	0.418	84.1 [69.9–93.4]	56.8 [41–71.7]	0.789 [0.656–0.844]	< 0.001
Peak IV	0.203	97.7 [88–99.9]	100 [92–100]	0.997 [0.953–1]	< 0.001
HAC-NR	0.477	95.5 [84.5–99.4]	86.4 [72.6–94.8]	0.965 [0.903–0.993]	< 0.001
Fi-SOLE	0.303	97.7 [88–99.9]	97.7 [88–99.9]	0.997 [0.954–1]	< 0.001

**Figure 5:**
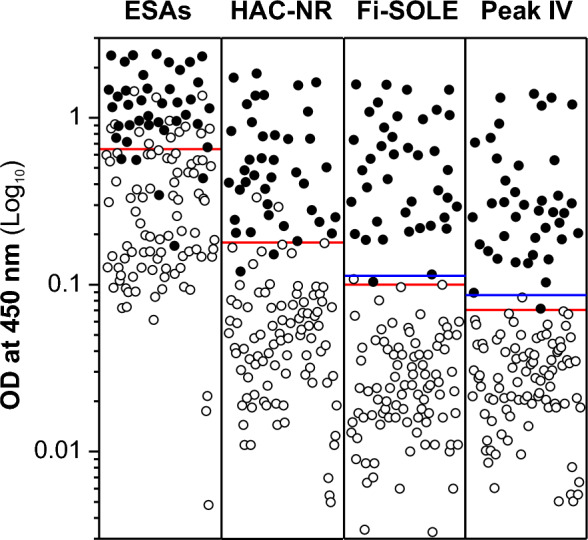
Plot showing serum IgG responses of infected (closed circles) and non-infected (open circles) sheep to four *F. hepatica* antigen preparations (ESAs, HAC-NR, Fi-SOLE and Peak IV). Each point represents an individual serum. The ELISA IgG values were expressed as Log_10_ OD response measured at 450 nm. Horizontal red bars represent optimal cut-off values obtained by ROC analysis for each antigen (see Table 2). Horizontal blue bars represent cut-off values obtained by ROC analysis selecting 100% specificity. The lowest and highest OD values obtained for each tested antigen were as follows: (i) sera from *Fasciola*-infected sheep: ESAs (0.17/2.40), HAC-NR (0.12/1.84), Fi-SOLE (0.10/1.58), Peak IV (0.07/1.31); (ii) sera from *Fasciola*-free sheep: ESAs (< 0.01/1.44), HAC-NR (< 0.01/0.33), Fi-SOLE (< 0.01/0.11), Peak IV (< 0.01/0.08).

**Figure 6:**
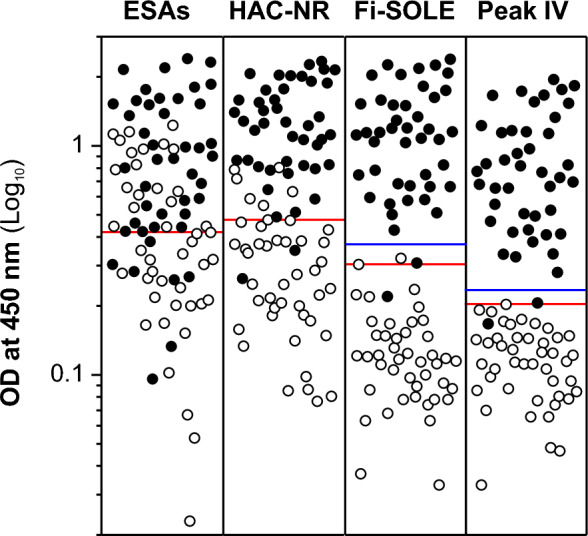
Plot showing serum IgG responses of infected (closed circles) and non-infected (open circles) cattle to four *F. hepatica* antigen preparations (ESAs, HAC-NR, Fi-SOLE and Peak IV). Each point represents an individual serum. The ELISA IgG values were expressed as Log_10_ OD response measured at 450 nm. Horizontal red bars represent optimal cut-off values obtained by ROC analysis for each antigen (see Table 3). Horizontal blue bars represent cut-off values obtained by ROC analysis selecting 100% specificity. The lowest and highest OD values obtained for each tested antigen were as follows: (i) sera from *Fasciola-*infected cattle: ESAs (0.1/2.40), HAC-NR (0.26/2.34), Fi-SOLE (0.22/2.39), Peak IV (0.17/1.95); (ii) sera from *Fasciola-*free cattle: ESAs (0.02/1.23), HAC-NR (0.08/0.81), Fi-SOLE (0.03/0.32), Peak IV (0.03/0.20).

The data in Figs. 5 and 6 showed the individual OD values obtained for each method after testing sera from infected (closed circles) and non-infected (open circles) sheep (Fig. 5) and cattle (Fig. 6). As can be observed, the OD values obtained for most sera from infected sheep and cattle were high for all tested antigens. Also, as expected, the observed OD values obtained for sera from non-infected sheep and cattle were much lower for purified antigens than for whole ESAs. In addition, when more conservative cut-off values were selected to ensure 100% specificity (see blue lines in Figs. 5 and 6), the sensitivities obtained for both antigens were also similar: 97.5% (Fi-SOLE and Peak IV) for ovine samples, and 95.5% (Fi-SOLE) and 97.7% (Peak IV), for bovine samples. The sensitivity and specificity values obtained for sheep and cattle with the antigenic fraction Fi-SOLE were like those previously reported for commercial devices using purified *Fasciola* ESAs^38^.

### Proteomic and immunological analysis of *Fasciola* ESAs fractions isolated by HAC and SEC

**Figure 7:**
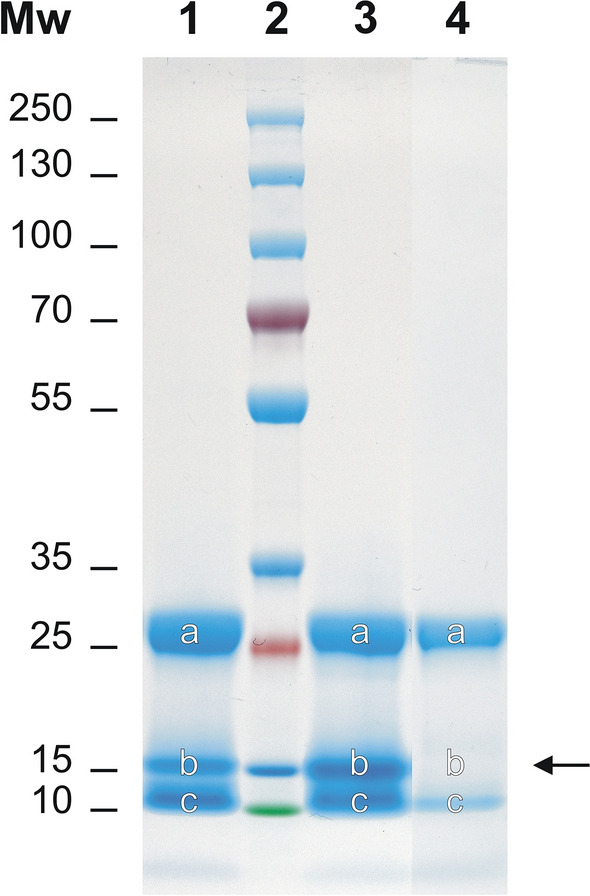
SDS-PAGE analysis of *F. hepatica* antigens in fraction HAC-NR (lane 1), Peak IV (lane 3) and fraction Fi-SOLE (lane 4). Lane 2 shows the isolation of the coloured molecular weight markers. The proteins in bands “a”, “b” and “c” were excised from the gel and analysed individually by MS/MS spectrometry. The arrow shows that band “b” in fraction Fi-SOLE (lane 4) was absent. A full view of the gel from which the bands of interest were excised for LC–MS/MS is shown in Fig. S1 (Supplementary file S1).

To identify the proteins in HAC-NR, Peak IV and Fi-SOLE fractions, we separated them in an SDS-PAGE gradient gel (8–16%) and the stained bands were scissed and processed for identification by nanoUHPLC-Tims-QTOF MS/MS spectrometry. The results of the SDS-PAGE study are showed in Fig. 7. As can be seen, most of the proteins belonging to fractions HAC-NR (lane 1) and Peak IV (lane 3) are grouped into three broad bands with MWs of 24–30 kDa (rows “a”), 14–17 kDa (rows “b”) and 10–13 kDa (rows “c”). The proteins of fraction Fi-SOLE (lane 4) presented the same pattern except that the band “b” was almost absent (see lane 4b, arrow). The nature and relative abundance of the proteins present in each SDS-PAGE band identified by mass spectrometry were summarized in Table 4 and Supplementary file S1. In Table 4, the identified proteins were grouped by categories (i.e., protein families) and spectral abundance (the number of spectra used to identify each protein), the latter been used here as a surrogate of the relative abundance of each protein according to the studies of Liu et al*.*^39^. It was reported that this method may have some shortcomings^40^, particularly when mass analysis is done under different experimental conditions, and since larger proteins contribute with more peptides than shorter ones. However, as we only compared peptides/proteins of similar molecular mass and all protein digestions and MS/MS analysis were done under the same experimental conditions, it is expected that our data accurately reflected the relative abundance of identified proteins. Single proteins, or protein families identified with a minimal of 15 spectra were annotated by their specific names in Table 4, while the remaining low-abundant proteins were included as a single miscellaneous group (others) within each category.

**Table 4:** Distribution and relative abundance of proteins identified by MS/MS spectrometry in *F. hepatica* antigen fractions (HAC-NR, Peak IV and Fi-SOLE) after SDS-PAGE analysis (see Fig. 7). (*) Only protein families identified by 15 or more spectra were individually represented. Individual proteins covering 5% or more of total spectra within each protein band were noted in bold. The total number of analyzed spectra was 4084 (HAC-NR), 3350 (Peak IV) and 814 (Fi-SOLE).

SDS-PAGE	HAC-NR (Lane 1)		PEAK IV (Lane 3)		Fi-SOLE (Lane 4)	
MW range	Protein family*	Spectra number (%)	Protein family*	Spectra number (%)	Protein family*	Spectra number (%)
24–30 kDa (bands “a”)	L-cathepsins	**1535** (95.3)	L-cathepsins	**947** (92.6)	L-cathepsins	**730** (99.9)
	Actins	16 (1.0)	GSTs	26 (2.5)	Others (n = 1)	1 (0.1)
	Others (n = 15)	59 (3.7)	Others (n = 14)	50 (4.9)		
14–17 kDa (bands “b”)	L-cathepsins	**486** (28.3)	FABPs	**665** (50.0)	Others (n = 3)	18 (100)
	FABPs	**447** (26.1)	Myoglobin	**236** (17.7)		
	Myoglobin	**279** (16.2)	Haemoglobin	**214** (16.1)		
	Haemoglobin	**238** (13.9)	L-cathepsins	**72** (5.4)		
	HSP-70	44 (2.6)	Globin-3	28 (2.1)		
	Thioredoxin	38 (2.2)	Others (n = 27)	115 (8.6)		
	Globin-3	32 (1.9)				
	Cystatin	20 (1.2)				
	SODs	16 (0.9)				
	Histones	15 (0.9)				
	Others (n = 20)	100 (5.8)				
10–13 kDa (bands “c”)	L-cathepsins	**307** (40.4)	FABPs	**301** (30.2)	L-cathepsins	**34** (53.3)
	Thioredoxin	**94** (12.4)	L-cathepsins	**193** (19.4)	Others (n = 12)	31 (47.7)
	Myoglobin	**75** (9.9)	Myoglobin	**112** (11.2)		
	Beta-tubulin	**47** (6.2)	Haemoglobin	**96** (9.6)		
	FABPs	**42** (5.5)	Thioredoxin	**84** (8.4)		
	Polyubiquitins	**40** (5.3)	Ca-binding proteins	**83** (8.3)		
	Cystatin	**40** (5.3)	Kunitz	20 (2.0)		
	Haemoglobin	34 (4.5)	Stefin-1	17 (1.7)		
	Others (n = 20)	80 (10.5)	Others (n = 23)	91 (9.1)		

When analysing the nature of proteins present in bands “a” (lanes 1, 3 and 4; Fig. 7), it was observed that most of them belong to the family of L-cathepsins, with relative abundances of 95.3% (HAC-NR), 92.6% (Peak IV) and 99.9% (Fi-SOLE). The protein content in bands “b” (lanes 1 and 3; Fig. 7) were more diverse, although four protein families predominated: L-cathepsin fragments, FABPs, myoglobin and haemoglobin. Among them, L-cathepsin fragments and FABPs were the most abundant proteins in the HAC-NR fraction (54.4%), while FABPs predominate in the Peak IV fraction (50.0%). In the SDS-PAGE region corresponding to lane 4b (Fi-SOLE) only small amounts of L-cathepsin fragments and cystatin were detected, all of them below the threshold of 15 spectra (Supplementary file S1), although this data should be taken with caution since the whole amount of protein in lane 4 was lower than in lanes 1 and 3. Regarding the proteins in bands “c” (lanes 1, 3 and 4; Fig. 7), fragments of L-cathepsins (40.4%), thioredoxin (12.7%) and myoglobin (9.9%) predominated in HAC-NR, while fragments of FABPs (30.2%), L-cathepsins (19.4%) and myoglobin (11.2%) were the most abundant in Peak IV. Fragments of L-cathepsins (53.3%) were also the major protein components of band “c” in fraction Fi-SOLE. Regarding the nature of the L-cathepsins present in the HAC-NR, Peak IV and Fi-SOLE fractions (Table 4), it should be noted that the MS/MS analysis mainly identified peptides belonging to FhCL1 > FhCL2 > FhCL5, but some peptides derived from FhCL3, FhCL4, FhCL6 and FhCL7 were also recognized (see Supplementary file S1). These results agreed with previous studies by Robinson et al.^41^ reporting that clades 1 and 2 account for 67.4 and 27.6% of total L-cathepsins secreted by adult flukes.

Comparing the diversity and predominance of proteins identified by MS/MS analysis of Peak IV, HAC-NR, and Fi-SOLE fractions, it would be expected that the Fi-SOLE fraction was the most specific in ELISA since this fraction is the one that contains the greater proportion of cathepsins and fragments thereof. However, at a first glance, neither the abundance of L-cathepsins nor the presence of other accompanying majority proteins could explain why the antigens in Peak-IV fraction are much more specific than those present in fraction HAC-NR (Table 4). So, to shed some light on this apparent contradiction, we first analysed which proteins identified by MS/MS analysis in the HAC-NR fraction (low specificity) were not present in either Peak-IV (highly specific) or Fi-SOLE (highly specific) fractions. The list of unique proteins present in fraction HAC-NR is showed in Table 5. As can be observed, β-tubulin > heat shock protein 70 (HSP-70) > actins > histones were the four most abundant proteins that were absent in Peak IV or Fi-SOLE fractions. These proteins are probably originated from the tegumental coat of the flukes, which is constantly released during the in vitro culture of flukes, and probably in more quantity when cultured for long time as with our ESAs preparation (24 h). Using a similar technology for protein identification (LC–MS/MS), the presence of actin, HSP-70, β-tubulin and other glycoproteins was already reported by Ravida et al*.*^11^ in a tegumental extract of *F. hepatica* obtained by treatment of adult flukes with Nonidet P-40 and further purification by lectin affinity chromatography.

**Table 5:** Diversity and relative abundance of unique proteins identified by MS/MS spectrometry in *F. hepatica* antigen in fraction HAC-NR which were not identified in fractions Fi-SOLE or Peak IV. Individual proteins covering 0.5% or more of total HAC-NR spectra (n = 4084) were noted in bold. The spectra number of unique proteins in fraction HAC-NR was n = 156.

Unique proteins	Spectra number	Percentage *
Actins [*Fasciola hepatica*]	**27**	**0.66**
Beta-tubulin [*Fasciola hepatica*]	**47**	**1.15**
Collagen type IV alpha 2 chain [*Fasciola hepatica*]	1	0.02
EGF region [*Fasciola hepatica*]	1	0.02
Fibrillin-2 [*Fasciola hepatica*]	1	0.02
Fructose-biphosphate aldolase B [*Fasciola hepatica*]	1	0.02
Heat shock protein 70 [*Fasciola hepatica*]	**44**	**1.08**
Histones [*Fasciola hepatica*]	**24**	**0.58**
Nuclear transport factor [*Fasciola hepatica*]	1	0.02
Tegumental CaBP4 [*Fasciola hepatica*]	6	0.15
Toll-interacting protein B [*Fasciola hepatica*]	1	0.02
Twitchin [*Fasciola hepatica*]	2	0.04

Anti-β-tubulin, anti-HSP-70, anti-actin and/or anti-histone antibodies have long been reported in serum of normal individuals^42,43^, but their presence is more frequently associated with autoimmune and inflammatory human disorders^44–47^. Likewise, anti-tubulin^48^, anti-HSP-70^49^ and anti-histone^50^ antibodies were also reported in serum from some animal species, but the available data on this topic are scarce. However, given the low proportion of these proteins in the HAC-NR antigenic fraction compared with the remaining proteins in such fraction (0.58–1.15%, see Table 5) it seems improbable that they were important cross-reactive target antigens responsible for the high background OD values observed with some serum samples obtained from non-infected cattle (see Fig. 6). Nevertheless, as the proteins included in Table 5 were not present in fractions Peak-IV or Fi-SOLE, we cannot completely rule out the possibility that one or more of such proteins could be targeted by antibodies from non-infected animals.

Regardless of proteins included in Table 5, there is also the possibility that antigens in the HAC-NR fraction were contaminated with foreign non-protein antigens (e.g., lipopolysaccharides, LPSs), or that some of them were decorated with ubiquitous cross-reacting epitopes (e.g., PC). LPSs (= endotoxins) constitute a family of structurally related glycolipids which are the major constituents of the outer membrane of most Gram-negative bacteria^51^. LPSs are frequent contaminants of laboratory preparations, and we have previously observed that *F. hepatica* ESAs may be contaminated with these molecules^10^. However, in this study we excluded that LPSs were present in the HAC-NR fraction for three reasons: (i) LPSs bind with high affinity to HA in the buffer used to obtain HAC-NR antigens^33,52^, (ii) the treatment of the HAC-NR fraction with allantoin crystalline powder (a recognized strong endotoxin adsorbent^53,54^) did not alter the antigenicity of HAC-NR fraction (data not shown) and (iii) LPSs are precipitable by ammonium sulphate^55^ under similar conditions as that used to obtain the specific antigenic fraction Fi-SOLE.

**Figure 8:**
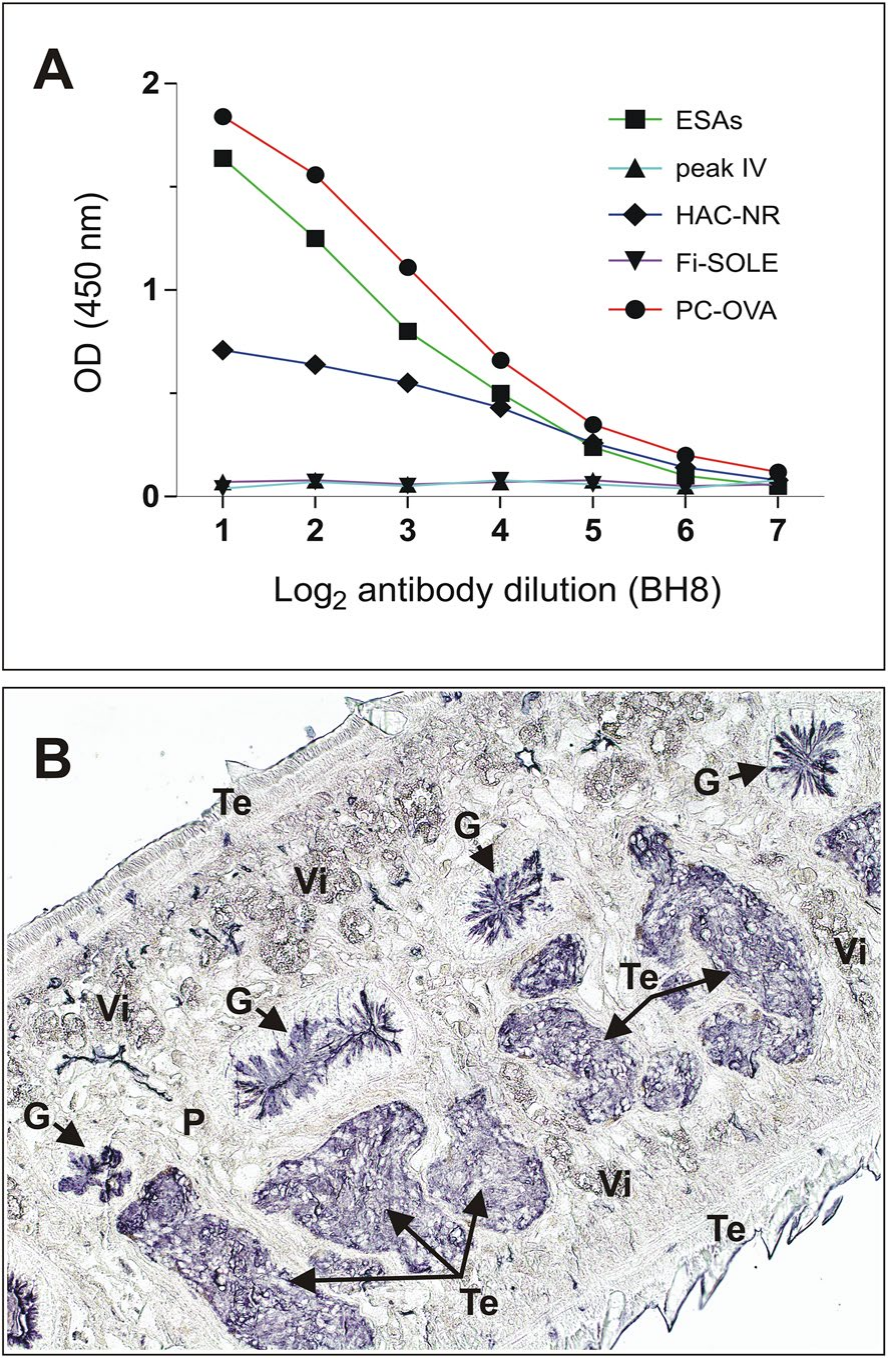
(**A**) Detection of PC-bearing antigens in four *F. hepatica* antigen fractions (ESAs, Peak IV, HAC-NR and Fi-SOLE) by iELISA. The graph shows the OD values obtained for several one-half serial dilutions of mAb BH8 (IgM anti-PC) starting at dilution 1/5000. PC-OVA was used as positive control. (**B**) Immunolocalization of PC-bearing antigens in adult flukes by immunoperoxidase staining of paraffin sections using mAb BH8 as probe. The low-magnification image shows positive staining of gut (G) and testes (Te) but not of parenchymal cells (P), vitelline glands (Vi) or tegument (Te).

PC is a small lipid-related hapten composed of a negatively charged phosphate and a positively charged choline group. PC is present in a wide/great variety of bacteria, fungi, protozoa, and helminths^56–59^. In general, PC can be found covalently attached to N-acetylglucosamine from N-glycans and glycolipids^60–62^ and is considered both a DAMP (danger-associated molecular pattern) and a PAMP (pathogen-associated molecular pattern)^63^. Early studies by Sloan *et al.*^64^ reported the presence of abundant immunodominant PC-bearing antigens in *F. hepatica* extracts, but the nature and anatomical location of them was never investigated. As the ELISA results presented above indicate that the specificity of the antigens contained in different fractions obtained from *F. hepatica* ESAs is different, we investigated the presence of PC-bearing antigens in such fractions. Accordingly, we measured the reactivity of seven two-fold serial dilutions of mAb BH8 (starting dilution 1/5000) against four ESAs fractions (whole ESAs, Peak IV, HAC-NR, Fi-SOLE) and PC-Ova (positive control) in iELISA. The data in Fig. 8A show that PC-bearing antigens were mainly present in whole ESAs followed by fraction HAC-NR, but not in the more specific Peak IV and Fi-SOLE fractions. These results suggest that most of the PC-bearing antigens in whole ESAs remain soluble in the filtrate after precipitation with 50% AS and filtration of the HAC-NR fraction (fraction Fi-NR, see Fig. 2). This hypothesis was later confirmed when we obtained strong reactivity of mAb BH8 with PBS-dialyzed antigens from the Fi-NR fraction in iELISA, but not against the HAC-RET antigens eluted with 0.5 M phosphate, which is recommended by the manufacturer to regenerate the HA column (not shown). However, as the protein concentration measured in Fi-NR was very low (see above), and no sugar traces could be detected using the phenol–sulphuric method^20^, knowing of the nature of the PC-bearing antigens in such fraction requires additional research.

The data of the IHQ presented in Fig. 8B also showed that, like L-cathepsins^24^, PC-bearing antigens are mainly present in the cecal epithelium, but also in other *Fasciola* structures as testes. The location of such antigens in the digestive tract of the parasite explains why PC-bearing antigens can be detected by mAb BH8 in ESAs after in vitro culture of the flukes. From these data, we tentatively suggest that PC-bearing molecules in ESAs may be involved in the cross-reactivity phenomenon observed when testing sera from some non-infected animals if whole ESAs are used as target in ELISA. Consistent with this hypothesis, preliminary ELISA inhibition studies in our laboratory showed that most of the cross-reactivity observed with sera from uninfected cattle can be eliminated by incorporating PC into the dilution buffer. Nevertheless, more studies will be required to definitively confirm this hypothesis and to know the nature, structure, and immunological relevance of the PC-bearing molecules detected in *F. hepatica* ESAs.

In summary, in this article we demonstrated that negative selection of *F. hepatica* ESAs by HAC combined with AS precipitation is a rapid, robust, cheap, and simple method to obtain an antigenic fraction rich in secreted native L-cathepsins. Purified antigens by this method can be used to improve the specificity of in-house ELISA methods targeting whole ESAs, but also to obtain antigens intended for the immunization of animals to produce anti-cathepsin L (mainly mixtures of CL1, CL2, CL5) polyclonal antibodies. The proposed method can also be used as a first step in the purification until homogeneity of *F. hepatica* L-cathepsins is achieved. Also, our study strongly suggest that PC-bearing molecules are major cross-reactive antigens present in *F. hepatica* ESAs. Although the obtention of purified native L-cathepsins from ESAs is limited by the availability of live adult flukes, and may be expensive and time-consuming, the methodology presented in this study may facilitate the task.

### Supplementary Information

Supplementary Information.

## Data Availability

The data from the current study are available from the corresponding author on reasonable request.
