# Supplementary material for: Increased specificity of Fasciola hepatica excretory-secretory antigens combining negative selection on hydroxyapatite and salt precipitation
Source: Sci Rep. 2024 Feb 16;14:3897. doi: 10.1038/s41598-024-54290-8 (PMC10873304; doi:10.1038/s41598-024-54290-8)

Increased specificity of *Fasciola hepatica* excretory-secretory antigens combining negative selection on hydroxyapatite and salt precipitation

Florencio M. Ubeira[1]*, Marta González-Warleta[2], Victoria Martínez-Sernández [1,3], José Antonio Castro-Hermida[2], Esperanza Paniagua[1], Fernanda Romarís[1] & Mercedes Mezo[2]

[1] Laboratorio de Parasitología, Facultad de Farmacia, 15782 Santiago de Compostela, Spain; Instituto de Investigación en Análisis Químicos y Biológicos (IAQBUS), Universidad de Santiago de Compostela, 15705 Santiago de Compostela, Spain.

[2] Laboratorio de Parasitología, Centro de Investigaciones Agrarias de Mabegondo, AGACAL, 15318 Abegondo (A Coruña), Spain.

[3] Servicio de Dermatología Médico-Quirúrgica y Venereología, Complejo Hospitalario Universitario de Pontevedra (CHUP), 36071 Pontevedra, Spain.

Table S1: List of proteins identified by nano-UHPLC-Tims-QTOF MS/MS analysis from the SDS-PAGE stained bands shown in Figure 7 of the HAC-NR, Peak IV, and Fi-SOLE fractions.

FIGURE 7, LANE 1A

Protein Group	Protein ID	Accession	-10lgP	Coverage (%)	Coverage (%) 1	Area 1	#Peptides	#Unique	#Spec 1	PTM	Avg. Mass	Description
1	1	ADP09371.1	179,39	33	33		19	0	93	Deamidation (NQ); Oxidation (M); Dehydration	36663	cathepsin L-like proteinase [Fasciola hepatica]
6	11	CAC12806.1	171,75	30	30	1,67E+03	18	1	84	Carbamidomethylation; Deamidation (NQ); Oxidation (M); Dehydration	35196	cathepsin L1 partial [Fasciola hepatica]
8	4	AAR99518.1	169,65	28	28		17	0	78	Carbamidomethylation; Deamidation (NQ); Oxidation (M); Dehydration	36714	cathepsin L protein [Fasciola hepatica]
3	5	AAM11647.1	169,46	31	31		17	0	89	Carbamidomethylation; Deamidation (NQ); Oxidation (M); Dehydration	35190	cathepsin L partial [Fasciola hepatica]
2	6	sp|Q24940.1|CATLL_FASHE	169,4	30	30		17	0	89	Carbamidomethylation; Deamidation (NQ); Oxidation (M); Dehydration	36896	RecName: Full=Cathepsin L-like proteinase; Flags: Precursor
2	7	AAA29136.1	169,4	30	30		17	0	89	Carbamidomethylation; Deamidation (NQ); Oxidation (M); Dehydration	36896	cathepsin [Fasciola hepatica]
4	12	CCA61803.1	168,71	29	29		17	0	89	Deamidation (NQ); Oxidation (M); Dehydration	36616	cathepsin protein CatL1-MM3p partial [Fasciola hepatica]
9	2	ACJ12894.1	168,46	30	30	0	16	2	76	Deamidation (NQ); Oxidation (M); Dehydration	36557	cathepsin L1D [Fasciola hepatica]
9	3	ACJ12893.1	168,46	30	30	0	16	2	76	Deamidation (NQ); Oxidation (M); Dehydration	36548	cathepsin L1D [Fasciola hepatica]
5	8	pdb|2O6X|A	164	33	33		15	0	85	Carbamidomethylation; Deamidation (NQ); Oxidation (M); Dehydration	35070	Chain A Secreted cathepsin L 1
5	9	AAB41670.2	164	32	32		15	0	85	Carbamidomethylation; Deamidation (NQ); Oxidation (M); Dehydration	36773	secreted cathepsin L 1 [Fasciola hepatica]
7	10	AAP49831.1	161,81	30	30	0	14	1	83	Carbamidomethylation; Deamidation (NQ); Oxidation (M); Dehydration	36602	cathepsin L partial [Fasciola hepatica]
10	14	AAR99519.1	159,44	36	36		17	0	71	Deamidation (NQ); Oxidation (M); Dehydration	26217	cathepsin L protein [Fasciola hepatica]
11	19	AAK38169.1	157,95	24	24		15	0	65	Deamidation (NQ); Oxidation (M); Dehydration	35234	cathepsin L-like partial [Fasciola hepatica]
12	22	BAA23743.1	147,51	24	24	0	13	1	61	Deamidation (NQ); Oxidation (M); Dehydration	36726	cathepsin L [Fasciola hepatica]
13	15	ATW63990.1	146,25	27	27	9,83E+03	10	3	44	Carbamidomethylation; Deamidation (NQ); Oxidation (M); Dehydration	35466	procathepsin L5 partial [Fasciola hepatica]
13	16	AAA29137.1	146,25	26	26	9,83E+03	10	3	44	Carbamidomethylation; Deamidation (NQ); Oxidation (M); Dehydration	37177	cathepsin [Fasciola hepatica]
13	17	AAF76330.1	146,25	26	26	9,83E+03	10	3	44	Carbamidomethylation; Deamidation (NQ); Oxidation (M); Dehydration	37149	cathepsin L [Fasciola hepatica]
21	24	THD25068.1	121,15	3	3	4,03E+02	8	8	10		269396	Filamin-A [Fasciola hepatica]
16	34	THD18939.1	114,02	19	19	0	7	1	21	Oxidation (M); Dehydration	26416	Secreted cathepsin L 1 [Fasciola hepatica]
19	26	ABG00259.1	109,11	21	21		7	0	12		24555	cathepsin L2 partial [Fasciola hepatica]
18	29	ASK40163.1	105,33	14	14		7	0	14		35438	L2 procathepsin partial [Fasciola hepatica]
18	30	AAC47721.1	105,33	14	14		7	0	14		37033	secreted cathepsin L 2 [Fasciola hepatica]
18	31	ABQ95351.1	105,33	14	14		7	0	14		37072	secreted cathepsin L2 [Fasciola hepatica]
18	32	CAA80446.1	105,33	14	14		7	0	14		37159	cathepsin L-like protease [Fasciola hepatica]
18	33	ABN50361.2	105,33	14	14		7	0	14		36981	cathepsin L [Fasciola hepatica]
15	42	THD18417.1	92,33	20	20		4	0	26	Deamidation (NQ)	14678	Cathepsin L [Fasciola hepatica]
25	47	THD25022.1	75,22	6	6	1,87E+03	3	3	6		30591	hypothetical protein D915_003906 [Fasciola hepatica]
27	39	THD18101.1	69,72	9	9		3	0	5	Oxidation (M)	37280	Cysteine protease [Fasciola hepatica]
17	48	THD23701.1	68,71	5	5		2	0	18	Dehydration	37063	Secreted cathepsin L 1 [Fasciola hepatica]
33	49	pdb|2VYW|A	67,5	13	13	0	2	2	3		16560	Chain A HEMOGLOBIN
33	50	THD25867.1	67,5	13	13	0	2	2	3		16691	Myoglobin 2 [Fasciola hepatica]
33	51	ABW96608.1	67,5	13	13	0	2	2	3		16691	hemoglobin F2 [Fasciola hepatica]
33	52	THD25868.1	67,5	13	13	0	2	2	3		16722	Myoglobin 2 [Fasciola hepatica]
24	66	THD18723.1	49,32	22	22		2	0	7		8691	putative pre-pro-cysteine proteinase [Fasciola hepatica]
31	61	THD27507.1	48,54	7	7	0	2	2	3		27325	Glutathione S-transferase omega class [Fasciola hepatica]
31	62	AFX98104.1	48,54	7	7	0	2	2	3		27334	glutathione S-transferase omega class [Fasciola hepatica]
29	67	ABZ80402.1	48,5	6	6	4,50E+03	2	1	3	Dehydration	34581	cathepsin L6 partial [Fasciola hepatica]
29	68	ABF85681.1	48,5	8	8	4,50E+03	2	1	3	Dehydration	26007	cathepsin L3 partial [Fasciola hepatica]
29	69	CAC12807.1	48,5	6	6	4,50E+03	2	1	3	Dehydration	35174	procathepsin L3 partial [Fasciola hepatica]
29	70	ABZ80399.1	48,5	6	6	4,50E+03	2	1	3	Dehydration	35333	cathepsin L3 partial [Fasciola hepatica]
29	71	ABW75768.2	48,5	5	5	4,50E+03	2	1	3	Dehydration	35718	procathepsin L partial [Fasciola hepatica]
29	72	QPX50259.1	48,5	5	5	4,50E+03	2	1	3	Dehydration	37306	cathepsin L3 peptidase [Fasciola hepatica]
29	73	ABW24657.1	48,5	5	5	4,50E+03	2	1	3	Dehydration	37418	cathepsin L [Fasciola hepatica]
29	74	ACM67633.1	48,5	5	5	4,50E+03	2	1	3	Dehydration	37216	cathepsin 1L [Fasciola hepatica]
29	75	ACM67632.1	48,5	5	5	4,50E+03	2	1	3	Dehydration	37455	cathepsin 2L [Fasciola hepatica]
30	93	THD18112.1	43,54	8	8	7,15E+03	1	1	3	Deamidation (NQ)	13016	Cathepsin L protease [Fasciola hepatica]
36	141	THD20839.1	42,41	4	4	0	2	2	2		66043	Cytoplasmic type actin 1 [Fasciola hepatica]
36	142	THD21966.1	42,41	7	7	0	2	2	2		41697	Cardiac muscle alpha actin [Fasciola hepatica]
36	143	THD24192.1	42,41	7	7	0	2	2	2		41772	Actin alpha cardiac muscle 1 [Fasciola hepatica]
36	144	THD24336.1	42,41	7	7	0	2	2	2		41758	Actin alpha cardiac muscle 1 [Fasciola hepatica]
36	145	THD22051.1	42,41	7	7	0	2	2	2		41646	Cytoplasmic actin [Fasciola hepatica]
36	146	THD25154.1	42,41	7	7	0	2	2	2		41772	Actin alpha cardiac muscle 1 [Fasciola hepatica]
36	147	THD26818.1	42,41	7	7	0	2	2	2		41786	Actin alpha cardiac muscle 1 [Fasciola hepatica]
36	148	THD24193.1	42,41	7	7	0	2	2	2		41758	Actin alpha cardiac muscle 1 [Fasciola hepatica]
42	95	THD19813.1	41,09	17	17	2,92E+03	1	1	1	Carbamidomethylation; Deamidation (NQ)	8669	Cathepsin L [Fasciola hepatica]
37	96	THD28418.1	40,52	4	4	0	1	1	2		20984	hypothetical protein D915_000689 [Fasciola hepatica]
32	77	THD24370.1	38,75	4	4	0	1	1	3	Oxidation (M)	25566	hypothetical protein D915_004622 [Fasciola hepatica]
43	103	THD23125.1	35,03	1	1	0	1	1	1		104439	Alpha actinin sarcomeric [Fasciola hepatica]
35	76	THD21169.1	33,54	8	8	0	1	1	2		11367	Histone H4 [Fasciola hepatica]
35	134	THD26313.1	33,54	11	11	0	1	1	2		8280	Histone H4 [Fasciola hepatica]
47	135	THD22536.1	33	3	3	0	1	1	1		39400	Fructose-bisphosphate aldolase B [Fasciola hepatica]

39	139	THD28845.1	32,03	0	0	0	1	1	1	Dehydration	703840	Twitchin [Fasciola hepatica]
44	78	THD20935.1	31,21	5	5	0	1	1	1		14635	Fatty acid binding protein a [Fasciola hepatica]
44	79	sp|Q9U1G6.1|FABP3_FASHE	31,21	5	5	0	1	1	1		14623	RecName: Full=Fatty acid-binding protein type 3
44	80	CAB65015.1	31,21	5	5	0	1	1	1		14623	fatty acid binding protein [Fasciola hepatica]
34	150	THD27851.1	30,51	1	1	1,69E+04	1	1	2		82596	T-cell immunomodulatory protein [Fasciola hepatica]
45	149	THD25210.1	30,12	2	2	0	1	1	1		47291	hypothetical protein D915_004034 [Fasciola hepatica]
41	151	THD21824.1	28,34	1	1	0	1	1	1		166419	Collagen type IV alpha 2 chain [Fasciola hepatica]
46	155	THD26223.1	27,63	7	7	0	1	1	1		14441	Histone H2A [Fasciola hepatica]
46	156	THD21594.1	27,63	7	7	0	1	1	1		13371	Histone H2A [Fasciola hepatica]
46	157	THD18428.1	27,63	7	7	0	1	1	1		13475	Histone H2A [Fasciola hepatica]
46	158	THD21592.1	27,63	7	7	0	1	1	1		13371	Histone H2A [Fasciola hepatica]
46	159	THD18298.1	27,63	7	7	0	1	1	1		13823	Histone H2A [Fasciola hepatica]
50	161	THD24294.1	26,8	1	1	1,01E+04	1	1	1	Deamidation (NQ)	59757	EGF region [Fasciola hepatica]

FIGURE 7, LANE 1B

Protein Group	Protein ID	Accession	-10lgP	Coverage (%)	Coverage (%) 2	Area 2	#Peptides	#Unique	#Spec 2	PTM	Avg. Mass	Description
1	12	THD25868.1	161,79	40	40	4,93E+04	10	2	135	Deamidation (NQ); De	16722	Myoglobin 2 [Fasciola hepatica]
2	13	pdb|2VYW|A	161,67	40	40	4,17E+03	10	2	119	Deamidation (NQ); De	16560	Chain A HEMOGLOBIN
2	14	THD25867.1	161,67	40	40	4,17E+03	10	2	119	Deamidation (NQ); De	16691	Myoglobin 2 [Fasciola hepatica]
2	15	ABW96608.1	161,67	40	40	4,17E+03	10	2	119	Deamidation (NQ); De	16691	hemoglobin F2 [Fasciola hepatica]
3	1	THD20935.1	160,66	66	66	3,59E+05	11	10	114	Carbamidomethylatio	14635	Fatty acid binding protein a [Fasciola hepatica]
3	2	sp|Q9U1G6.1|FABP3_FASHE	160,66	66	66	3,59E+05	11	10	114	Carbamidomethylatio	14623	RecName: Full=Fatty acid-binding protein type 3
3	3	CAB65015.1	160,66	66	66	3,59E+05	11	10	114	Carbamidomethylatio	14623	fatty acid binding protein [Fasciola hepatica]
4	4	CAC12806.1	147,41	24	24		12	0	53	Oxidation (M)	35196	cathepsin L1 partial [Fasciola hepatica]
9	26	THD25871.1	146,04	34	34	1,49E+04	8	5	32	Deamidation (NQ); Ox	17084	Globin-3 [Fasciola hepatica]
5	9	AAR99518.1	141,39	22	22		10	0	46	Oxidation (M)	36714	cathepsin L protein [Fasciola hepatica]
7	6	ATW63990.1	139,04	22	22	0	8	1	39	Carbamidomethylatio	35466	procathepsin L5 partial [Fasciola hepatica]
7	7	AAA29137.1	139,04	21	21	0	8	1	39	Carbamidomethylatio	37177	cathepsin [Fasciola hepatica]
7	8	AAF76330.1	139,04	21	21	0	8	1	39	Carbamidomethylatio	37149	cathepsin L [Fasciola hepatica]
6	10	THD25753.1	137,14	55	55	6,14E+04	9	8	43	Acetylation (Protein N	14936	Fatty acid-binding protein type V [Fasciola hepatica]
6	11	sp|Q7M4G1.2|FABP2_FASHE	137,14	55	55	6,14E+04	9	8	43	Acetylation (Protein N	14936	RecName: Full=Fatty acid-binding protein type 2
10	16	ADP09371.1	136,81	21	21		10	0	31	Oxidation (M)	36663	cathepsin L-like proteinase [Fasciola hepatica]
8	22	ACJ12893.1	124,23	21	21	0	9	1	33	Oxidation (M)	36548	cathepsin L1D [Fasciola hepatica]
8	23	ACJ12894.1	124,23	21	21	0	9	1	33	Oxidation (M)	36557	cathepsin L1D [Fasciola hepatica]
8	24	QIB89441.1	124,23	11	11	0	9	1	33	Oxidation (M)	69865	cathepsin L7 [Fasciola hepatica]
12	27	AAP49831.1	120,61	19	19	0	8	1	27	Oxidation (M); Dehyd	36602	cathepsin L partial [Fasciola hepatica]
13	30	THD25869.1	117,37	41	41	4,31E+04	6	5	25	Deamidation (NQ)	14232	Myoglobin 2 [Fasciola hepatica]
11	38	AAR99519.1	115,09	23	23		9	0	31	Oxidation (M)	26217	cathepsin L protein [Fasciola hepatica]
16	36	CAB65014.1	109,36	51	51	1,45E+04	7	7	19	Deamidation (NQ)	11683	thioredoxin (TRX) [Fasciola hepatica]
16	37	pdb|2VIM|A	109,36	51	51	1,45E+04	7	7	19	Deamidation (NQ)	11683	Chain A Thioredoxin
19	68	THD25870.1	99,08	30	30	7,96E+03	4	2	12	Oxidation (M)	8022	hypothetical protein D915_003343 [Fasciola hepatica]
21	31	THD25273.1	94,76	4	4	1,06E+03	6	6	11	Oxidation (M)	171980	Multi-domain cystatin [Fasciola hepatica]
24	45	THD25752.1	94,25	30	30	0	4	3	8	Acetylation (Protein N	14712	Fatty acid-binding protein type 2 [Fasciola hepatica]
24	46	sp|Q7M4G0.3|FABP1_FASHE	94,25	30	30	0	4	3	8	Acetylation (Protein N	14712	RecName: Full=Fatty acid-binding protein Fh15
22	32	ASK40163.1	93,76	16	16	0	6	3	9		35438	L2 procathepsin partial [Fasciola hepatica]
22	33	AAC47721.1	93,76	15	15	0	6	3	9		37033	secreted cathepsin L 2 [Fasciola hepatica]
22	34	ABN50361.2	93,76	15	15	0	6	3	9		36981	cathepsin L [Fasciola hepatica]
22	35	ABQ95351.1	93,76	15	15	0	6	3	9		37072	secreted cathepsin L2 [Fasciola hepatica]
18	50	THD18939.1	93,49	16	16	0	4	1	15	Oxidation (M)	26416	Secreted cathepsin L 1 [Fasciola hepatica]
23	53	THD25954.1	91,71	23	23	1,37E+03	3	3	8	Oxidation (M)	15826	Superoxide dismutase [Fasciola hepatica]
23	54	AAD30361.1	91,71	25	25	1,37E+03	3	3	8	Oxidation (M)	14979	Cu/Zn-superoxide dismutase [Fasciola hepatica]
15	59	THD18417.1	88,12	18	18	0	3	1	21		14678	Cathepsin L [Fasciola hepatica]
36	57	THD28453.1	82,46	4	4	8,15E+02	3	3	3		83852	Titin [Fasciola hepatica]
30	47	THD25068.1	80,16	2	2	0	4	4	4		269396	Filamin-A [Fasciola hepatica]
33	87	THD23439.1	70,12	9	9	0	2	2	4	Deamidation (NQ); Ox	17732	Peptidyl-prolyl cis-trans isomerase [Fasciola hepatica]
45	64	THD25658.1	66,65	4	4	0	2	2	2	Oxidation (M)	73844	L-plastin [Fasciola hepatica]
25	73	THD18299.1	64,28	18	18	0	3	3	7	Oxidation (M)	12063	Mitochondrial cytochrome c [Fasciola hepatica]
38	77	pdb|6I1M|A	56,8	22	22	0	2	2	3	Oxidation (M)	9792	Chain A Cystatin
38	78	THD19412.1	56,8	17	17	0	2	2	3	Oxidation (M)	12490	Type-1 cystatin cysteine protease inhibitor [Fasciola hepatica]
38	79	AAV68752.1	56,8	17	17	0	2	2	3	Oxidation (M)	12472	cystatin [Fasciola hepatica]
50	84	THD24007.1	54,88	12	12	0	2	2	2		17889	Myophilin [Fasciola hepatica]
32	81	ABZ80401.1	54,38	6	6	0	2	1	4	Dehydration	34274	cathepsin L4 partial [Fasciola hepatica]
32	82	ABZ80400.1	54,38	6	6	0	2	1	4	Dehydration	34128	cathepsin L4 partial [Fasciola hepatica]
29	75	THD22756.1	53,86	9	9	2,76E+03	2	2	5		24823	hypothetical protein D915_006522 [Fasciola hepatica]
34	129	THD19782.1	51,89	5	5	4,21E+03	1	1	4		18531	hypothetical protein D915_009347 [Fasciola hepatica]
60	130	THD18112.1	50,06	8	8	0	1	1	1		13016	Cathepsin L protease [Fasciola hepatica]
35	63	THD25754.1	49,8	20	20	0	3	2	3	Acetylation (Protein N	14760	Fatty acid-binding protein type 3 [Fasciola hepatica]
51	131	THD19897.1	48,25	10	10	8,11E+02	1	1	2		11069	Stefin-1 [Fasciola hepatica]
41	85	THD21681.1	47,49	15	15	0	2	2	3		17348	Calmodulin/ calcium-binding protein/ EF-Hand superfamily protein [Fasciola hepatica]
41	86	CAL91033.1	47,49	15	15	0	2	2	3		17348	calmodulin-like protein 2 (CaM2) [Fasciola hepatica]
47	83	THD21169.1	42,92	17	17	0	2	2	2		11367	Histone H4 [Fasciola hepatica]
27	127	AIE76459.1	42,89	8	8	1,69E+03	1	1	6		14077	CD59-like protein [Fasciola hepatica]
27	128	THD28115.1	42,89	8	8	1,69E+03	1	1	6		14077	CD59 protein [Fasciola hepatica]
42	140	THD17940.1	41,85	12	12	1,66E+03	1	1	3		10871	Titin partial [Fasciola hepatica]
46	121	THD27507.1	41,52	7	7	0	2	2	2		27325	Glutathione S-transferase omega class [Fasciola hepatica]
46	122	AFX98104.1	41,52	7	7	0	2	2	2		27334	glutathione S-transferase omega class [Fasciola hepatica]
44	98	QIS63047.1	40,41	7	7	0	2	2	2		24323	heat shock protein 70 partial [Fasciola hepatica]
44	99	QIS63043.1	40,41	7	7	0	2	2	2		24323	heat shock protein 70 partial [Fasciola hepatica]

44	100	QIS63036.1	40,41	7	7	0	2	2	2		24323	heat shock protein 70 partial [Fasciola hepatica]
44	101	QIS63032.1	40,41	7	7	0	2	2	2		24323	heat shock protein 70 partial [Fasciola hepatica]
44	102	QIS63044.1	40,41	7	7	0	2	2	2		24323	heat shock protein 70 partial [Fasciola hepatica]
44	103	QIS63033.1	40,41	7	7	0	2	2	2		24323	heat shock protein 70 partial [Fasciola hepatica]
44	104	QIS63035.1	40,41	7	7	0	2	2	2		24323	heat shock protein 70 partial [Fasciola hepatica]
44	105	QIS63037.1	40,41	7	7	0	2	2	2		24323	heat shock protein 70 partial [Fasciola hepatica]
44	106	QIS63042.1	40,41	7	7	0	2	2	2		24323	heat shock protein 70 partial [Fasciola hepatica]
44	107	QIS63039.1	40,41	7	7	0	2	2	2		24323	heat shock protein 70 partial [Fasciola hepatica]
44	108	QIS63040.1	40,41	7	7	0	2	2	2		24323	heat shock protein 70 partial [Fasciola hepatica]
44	109	QIS63064.1	40,41	7	7	0	2	2	2		24323	heat shock protein 70 partial [Fasciola hepatica]
44	110	QIS76930.1	40,41	7	7	0	2	2	2		24323	heat shock protein 70 partial [Fasciola hepatica]
44	111	QIS63031.1	40,41	7	7	0	2	2	2		24323	heat shock protein 70 partial [Fasciola hepatica]
44	112	QIS63034.1	40,41	7	7	0	2	2	2		24323	heat shock protein 70 partial [Fasciola hepatica]
44	113	QIS63045.1	40,41	7	7	0	2	2	2		24323	heat shock protein 70 partial [Fasciola hepatica]
44	114	QIS63046.1	40,41	7	7	0	2	2	2		24323	heat shock protein 70 partial [Fasciola hepatica]
44	115	QIS63048.1	40,41	7	7	0	2	2	2		24323	heat shock protein 70 partial [Fasciola hepatica]
44	116	QIS63041.1	40,41	7	7	0	2	2	2		24323	heat shock protein 70 partial [Fasciola hepatica]
44	117	QIS63038.1	40,41	7	7	0	2	2	2		24323	heat shock protein 70 partial [Fasciola hepatica]
44	118	THD28020.1	40,41	2	2	0	2	2	2		70753	Major heat shock 70 kDa protein Bbb [Fasciola hepatica]
44	119	ABS52704.1	40,41	2	2	0	2	2	2		70753	heat shock protein 70 [Fasciola hepatica]
55	138	THD27289.1	40,33	4	4	0	1	1	1		29334	Toll-interacting protein B [Fasciola hepatica]
61	142	THD27119.1	38,37	2	2	0	1	1	1		72518	Fasciclin-1 [Fasciola hepatica]
43	124	THD28845.1	36,6	0	0	0	1	1	2		703840	Twitchin [Fasciola hepatica]
48	146	THD21594.1	34,41	7	7	0	1	1	2		13371	Histone H2A [Fasciola hepatica]
48	147	THD21592.1	34,41	7	7	0	1	1	2		13371	Histone H2A [Fasciola hepatica]
48	148	THD18428.1	34,41	7	7	0	1	1	2		13475	Histone H2A [Fasciola hepatica]
48	149	THD18298.1	34,41	7	7	0	1	1	2		13823	Histone H2A [Fasciola hepatica]
48	150	THD26223.1	34,41	7	7	0	1	1	2		14441	Histone H2A [Fasciola hepatica]
56	151	THD24822.1	33,82	1	1	1,19E+03	1	1	1	Deamidation (NQ)	92519	Sodium and chloride dependent glycine [Fasciola hepatica]
39	175	AAB46830.1	30,58	12	12	0	1	1	3		6589	Fh-KTM=6.751 kda monomeric Kunitz-type proteinase inhibitor [Fasciola hepatica=trematode Peptide 58 aa]
39	176	THD28325.1	30,58	8	8	0	1	1	3		9314	Kunitz-CH [Fasciola hepatica]
39	177	CEL12048.1	30,58	8	8	0	1	1	3		9252	Kunitz [Fasciola hepatica]
39	178	CEL12049.1	30,58	8	8	0	1	1	3		9356	Kunitz-CH [Fasciola hepatica]
57	180	THD25594.1	28,93	0	0	0	1	1	1		284868	Filamin-A [Fasciola hepatica]
49	181	THD28589.1	28,8	0	0	0	1	1	2		190619	Tensin [Fasciola hepatica]
64	182	THD19813.1	27,97	17	17	0	1	1	1	Carbamidomethylatio	8669	Cathepsin L [Fasciola hepatica]
65	191	THD19210.1	27,01	7	7	0	1	1	1		13570	Histone H2B [Fasciola hepatica]
65	192	THD18062.1	27,01	7	7	0	1	1	1		13677	Histone H2B [Fasciola hepatica]
65	193	THD21593.1	27,01	7	7	0	1	1	1		13677	Histone H2B [Fasciola hepatica]
54	183	THD24192.1	26,94	4	4	0	1	1	1		41772	Actin alpha cardiac muscle 1 [Fasciola hepatica]
54	184	THD24336.1	26,94	4	4	0	1	1	1		41758	Actin alpha cardiac muscle 1 [Fasciola hepatica]
54	185	THD22051.1	26,94	4	4	0	1	1	1		41646	Cytoplasmic actin [Fasciola hepatica]
54	186	THD25154.1	26,94	4	4	0	1	1	1		41772	Actin alpha cardiac muscle 1 [Fasciola hepatica]
54	187	THD26818.1	26,94	4	4	0	1	1	1		41786	Actin alpha cardiac muscle 1 [Fasciola hepatica]
54	188	THD24193.1	26,94	4	4	0	1	1	1		41758	Actin alpha cardiac muscle 1 [Fasciola hepatica]
54	189	THD21966.1	26,94	4	4	0	1	1	1		41697	Cardiac muscle alpha actin [Fasciola hepatica]
54	194	THD20839.1	26,94	3	3	0	1	1	1		66043	Cytoplasmic type actin 1 [Fasciola hepatica]
52	200	THD27851.1	23,81	1	1	0	1	1	1		82596	T-cell immunomodulatory protein [Fasciola hepatica]
59	204	THD26611.1	22,88	1	1	0	1	1	1		110171	Titin [Fasciola hepatica]

FIGURE 7, LANE 1C

Protein Group	Protein ID	Accession	-10lgP	Coverage (%)	Coverage (%) 3	Area 3	#Peptides	#Unique	#Spec 3	PTM	Avg. Mass	Description
5	9	AAR99518.1	130,19	17	17		9	0	24		36714	cathepsin L protein [Fasciola hepatica]
3	13	pdb|2VYW|A	129,94	32	32	0	8	2	34		16560	Chain A HEMOGLOBIN
3	14	THD25867.1	129,94	32	32	0	8	2	34		16691	Myoglobin 2 [Fasciola hepatica]
3	15	ABW96608.1	129,94	32	32	0	8	2	34		16691	hemoglobin F2 [Fasciola hepatica]
2	12	THD25868.1	129,66	32	32	8,17E+03	8	2	35		16722	Myoglobin 2 [Fasciola hepatica]
6	17	AAM11647.1	128,35	18	18	0	9	1	23		35190	cathepsin L partial [Fasciola hepatica]
6	19	AAA29136.1	128,35	17	17	0	9	1	23		36896	cathepsin [Fasciola hepatica]
6	20	sp|Q24940.1|CATLL_FASHE	128,35	17	17	0	9	1	23		36896	RecName: Full=Cathepsin L-like proteinase; Flags: Precursor
6	18	CCA61803.1	128,35	17	17	0	9	1	23		36616	cathepsin protein CatL1-MM3p partial [Fasciola hepatica]
6	16	ADP09371.1	128,35	17	17	0	9	1	23		36663	cathepsin L-like proteinase [Fasciola hepatica]
9	6	ATW63990.1	127,74	19	19	2,47E+02	6	1	19	Carbamidomethylation	35466	procathepsin L5 partial [Fasciola hepatica]
9	7	AAA29137.1	127,74	18	18	2,47E+02	6	1	19	Carbamidomethylation	37177	cathepsin [Fasciola hepatica]
9	8	AAF76330.1	127,74	18	18	2,47E+02	6	1	19	Carbamidomethylation	37149	cathepsin L [Fasciola hepatica]
7	4	CAC12806.1	125,49	18	18	2,45E+04	9	2	22		35196	cathepsin L1 partial [Fasciola hepatica]
1	36	CAB65014.1	125,02	59	59	9,07E+04	8	8	47	Deamidation (NQ)	11683	thioredoxin (TRX) [Fasciola hepatica]
1	37	pdb|2VIM|A	125,02	59	59	9,07E+04	8	8	47	Deamidation (NQ)	11683	Chain A Thioredoxin
11	1	THD20935.1	108,45	36	36	1,25E+04	6	6	10	Oxidation (M)	14635	Fatty acid binding protein a [Fasciola hepatica]
11	2	sp|Q9U1G6.1|FABP3_FASHE	108,45	36	36	1,25E+04	6	6	10	Oxidation (M)	14623	RecName: Full=Fatty acid-binding protein type 3
11	3	CAB65015.1	108,45	36	36	1,25E+04	6	6	10	Oxidation (M)	14623	fatty acid binding protein [Fasciola hepatica]
13	131	THD19897.1	99,26	29	29	6,80E+03	5	5	9		11069	Stefin-1 [Fasciola hepatica]
8	6224	THD19920.1	97,12	20	20	5,47E+03	4	4	20		17188	Polyubiquitin-B [Fasciola hepatica]
8	6225	THD18230.1	97,12	8	8	5,47E+03	4	4	20		42830	Polyubiquitin-C [Fasciola hepatica]
12	32	ASK40163.1	94,93	12	12	0	4	1	8		35438	L2 procathepsin partial [Fasciola hepatica]
12	33	AAC47721.1	94,93	11	11	0	4	1	8		37033	secreted cathepsin L 2 [Fasciola hepatica]
12	34	ABN50361.2	94,93	11	11	0	4	1	8		36981	cathepsin L [Fasciola hepatica]
12	35	ABQ95351.1	94,93	11	11	0	4	1	8		37072	secreted cathepsin L2 [Fasciola hepatica]
15	30	THD25869.1	90,47	33	33	1,88E+03	4	3	6		14232	Myoglobin 2 [Fasciola hepatica]
10	78	THD19412.1	88,89	33	33	1,81E+04	4	4	18	Oxidation (M)	12490	Type-1 cystatin cysteine protease inhibitor [Fasciola hepatica]
10	79	AAV68752.1	88,89	33	33	1,81E+04	4	4	18	Oxidation (M)	12472	cystatin [Fasciola hepatica]
16	26	THD25871.1	84,27	28	28	2,46E+03	4	3	6	Deamidation (NQ)	17084	Globin-3 [Fasciola hepatica]
20	87	THD23439.1	84,14	13	13	1,36E+03	3	3	4	Oxidation (M)	17732	Peptidyl-prolyl cis-trans isomerase [Fasciola hepatica]
18	6226	THD25975.1	79,28	17	17	0	4	4	6	Oxidation (M)	22035	Tegumental calcium-binding EF-hand protein 4 [Fasciola hepatica]
18	6227	CAA06036.1	79,28	17	17	0	4	4	6	Oxidation (M)	22028	calcium-binding protein [Fasciola hepatica]
18	6228	AML33332.1	79,28	17	17	0	4	4	6	Oxidation (M)	22035	CaBP1 [Fasciola hepatica]
14	59	THD18417.1	78,82	20	20	0	3	1	7		14678	Cathepsin L [Fasciola hepatica]
17	10	THD25753.1	77,66	22	22	9,51E+02	3	3	6	Oxidation (M)	14936	Fatty acid-binding protein type V [Fasciola hepatica]
17	11	sp|Q7M4G1.2|FABP2_FASHE	77,66	22	22	9,51E+02	3	3	6	Oxidation (M)	14936	RecName: Full=Fatty acid-binding protein type 2
23	68	THD25870.1	66,51	16	16	7,33E+02	2	2	3		8022	hypothetical protein D915_003343 [Fasciola hepatica]
22	197	ABZ80402.1	63,78	6	6		2	0	4	Dehydration	34581	cathepsin L6 partial [Fasciola hepatica]
19	31	THD25273.1	63,37	1	1	0	2	2	4		171980	Multi-domain cystatin [Fasciola hepatica]
32	57	THD28453.1	60,09	2	2	4,84E+02	1	1	1		83852	Titin [Fasciola hepatica]
21	123	ABZ80399.1	52,9	6	6		2	0	4	Dehydration	35333	cathepsin L3 partial [Fasciola hepatica]
21	125	ACM67632.1	52,9	5	5		2	0	4	Dehydration	37455	cathepsin 2L [Fasciola hepatica]
33	185	THD22051.1	52,7	4	4	0	1	1	1		41646	Cytoplasmic actin [Fasciola hepatica]
33	189	THD21966.1	52,7	4	4	0	1	1	1		41697	Cardiac muscle alpha actin [Fasciola hepatica]
33	183	THD24192.1	52,7	4	4	0	1	1	1		41772	Actin alpha cardiac muscle 1 [Fasciola hepatica]
33	184	THD24336.1	52,7	4	4	0	1	1	1		41758	Actin alpha cardiac muscle 1 [Fasciola hepatica]
33	186	THD25154.1	52,7	4	4	0	1	1	1		41772	Actin alpha cardiac muscle 1 [Fasciola hepatica]
33	187	THD26818.1	52,7	4	4	0	1	1	1		41786	Actin alpha cardiac muscle 1 [Fasciola hepatica]
33	188	THD24193.1	52,7	4	4	0	1	1	1		41758	Actin alpha cardiac muscle 1 [Fasciola hepatica]
33	194	THD20839.1	52,7	3	3	0	1	1	1		66043	Cytoplasmic type actin 1 [Fasciola hepatica]
28	73	THD18299.1	44,77	10	10	0	1	1	2		12063	Mitochondrial cytochrome c [Fasciola hepatica]
40	6235	THD21853.1	44,4	9	9	0	1	1	1	Oxidation (M)	14124	Nuclear transport factor 2 [Fasciola hepatica]
29	130	THD18112.1	41,55	8	8	0	1	1	2		13016	Cathepsin L protease [Fasciola hepatica]
24	127	AIE76459.1	40,5	8	8	0	1	1	3		14077	CD59-like protein [Fasciola hepatica]
24	128	THD28115.1	40,5	8	8	0	1	1	3		14077	CD59 protein [Fasciola hepatica]
30	182	THD19813.1	39,99	17	17	1,69E+03	1	1	2	Carbamidomethylation; Deamidation (NQ)	8669	Cathepsin L [Fasciola hepatica]
31	209	THD21260.1	37,63	1	1	0	1	1	2		80464	von Willebrand factor A domain-containing protein 5A [Fasciola hepatica]
41	6236	THD24513.1	37,07	2	2	0	1	1	1	Oxidation (M)	78182	Enamine/imine deaminase [Fasciola hepatica]
42	142	THD27119.1	36,02	2	2	0	1	1	1		72518	Fasciclin-1 [Fasciola hepatica]
43	6237	THD28916.1	35,81	12	12	0	1	1	1	Oxidation (M)	9818	hypothetical protein D915_000254 [Fasciola hepatica]
37	6234	THD23562.1	35,37	2	2	0	1	1	1		57624	Ubiquilin [Fasciola hepatica]
25	175	AAB46830.1	35,22	12	12	5,70E+02	1	1	2		6589	Fh-KTM=6.751 kda monomeric Kunitz-type proteinase inhibitor [Fasciola hepatica=trematode Peptide 58 aa]
25	177	CEL12048.1	35,22	8	8	5,70E+02	1	1	2		9252	Kunitz [Fasciola hepatica]
25	176	THD28325.1	35,22	8	8	5,70E+02	1	1	2		9314	Kunitz-CH [Fasciola hepatica]
25	178	CEL12049.1	35,22	8	8	5,70E+02	1	1	2		9356	Kunitz-CH [Fasciola hepatica]
38	2344	QHB80625.1	33,84	5	5	0	1	1	1		30946	beta-tubulin partial [Fasciola hepatica]
38	2350	QHB80669.1	33,84	4	4	0	1	1	1		39200	beta-tubulin partial [Fasciola hepatica]
38	2351	QHB80635.1	33,84	4	4	0	1	1	1		40416	beta-tubulin partial [Fasciola hepatica]

38	2352	QHB80658.1	33,84	4	4	0	1	1	1		41041	beta-tubulin partial [Fasciola hepatica]
38	2353	QHB80634.1	33,84	4	4	0	1	1	1		40714	beta-tubulin partial [Fasciola hepatica]
38	2355	QHB80646.1	33,84	4	4	0	1	1	1		42744	beta-tubulin partial [Fasciola hepatica]
38	2356	QHB80668.1	33,84	4	4	0	1	1	1		42744	beta-tubulin partial [Fasciola hepatica]
38	2357	QHB80645.1	33,84	4	4	0	1	1	1		42872	beta-tubulin partial [Fasciola hepatica]
38	2358	QHB80613.1	33,84	4	4	0	1	1	1		43151	beta-tubulin partial [Fasciola hepatica]
38	2359	QHB80678.1	33,84	4	4	0	1	1	1		43324	beta-tubulin partial [Fasciola hepatica]
38	2360	QHB80647.1	33,84	4	4	0	1	1	1		43514	beta-tubulin partial [Fasciola hepatica]
38	2361	QHB80636.1	33,84	4	4	0	1	1	1		43514	beta-tubulin partial [Fasciola hepatica]
38	2362	QHB80614.1	33,84	4	4	0	1	1	1		43614	beta-tubulin partial [Fasciola hepatica]
38	2363	QHB80612.1	33,84	4	4	0	1	1	1		43455	beta-tubulin partial [Fasciola hepatica]
38	2364	QHB80623.1	33,84	4	4	0	1	1	1		43783	beta-tubulin partial [Fasciola hepatica]
38	2365	QHB80657.1	33,84	4	4	0	1	1	1		43815	beta-tubulin partial [Fasciola hepatica]
38	2366	QHB80690.1	33,84	4	4	0	1	1	1		43976	beta-tubulin partial [Fasciola hepatica]
38	2367	QHB80667.1	33,84	4	4	0	1	1	1		44162	beta-tubulin partial [Fasciola hepatica]
38	2368	QHB80624.1	33,84	4	4	0	1	1	1		44245	beta-tubulin partial [Fasciola hepatica]
38	2369	QHB80679.1	33,84	4	4	0	1	1	1		44245	beta-tubulin partial [Fasciola hepatica]
38	2370	QHB80680.1	33,84	4	4	0	1	1	1		44417	beta-tubulin partial [Fasciola hepatica]
38	2371	QHB80689.1	33,84	4	4	0	1	1	1		44290	beta-tubulin partial [Fasciola hepatica]
38	2372	QHB80656.1	33,84	4	4	0	1	1	1		44281	beta-tubulin partial [Fasciola hepatica]
38	2373	QHB80633.1	33,84	4	4	0	1	1	1		44433	beta-tubulin partial [Fasciola hepatica]
38	2374	QHB80666.1	33,84	4	4	0	1	1	1		44433	beta-tubulin partial [Fasciola hepatica]
38	2375	QHB80644.1	33,84	4	4	0	1	1	1		44548	beta-tubulin partial [Fasciola hepatica]
38	2376	QHB80655.1	33,84	4	4	0	1	1	1		44644	beta-tubulin partial [Fasciola hepatica]
38	2377	QHB80688.1	33,84	3	3	0	1	1	1		44977	beta-tubulin partial [Fasciola hepatica]
38	2378	QHB80622.1	33,84	3	3	0	1	1	1		44977	beta-tubulin partial [Fasciola hepatica]
38	2379	QHB80677.1	33,84	3	3	0	1	1	1		45268	beta-tubulin partial [Fasciola hepatica]
38	2380	QHB80611.1	33,84	3	3	0	1	1	1		45268	beta-tubulin partial [Fasciola hepatica]
38	2383	CAC82577.1	33,84	3	3	0	1	1	1		48859	beta-tubulin partial [Fasciola hepatica]
38	3432	THD28242.1	33,84	3	3	0	1	1	1		49369	Tubulin beta chain [Fasciola hepatica]
38	2384	THD27772.1	33,84	3	3	0	1	1	1		49815	Tubulin beta chain [Fasciola hepatica]
38	2385	CAO79610.1	33,84	3	3	0	1	1	1		49821	beta-tubulin [Fasciola hepatica]
38	2386	CAP72052.1	33,84	3	3	0	1	1	1		49838	tubulin beta-4 [Fasciola hepatica]
38	6238	THD28586.1	33,84	3	3	0	1	1	1		49623	Tubulin beta chain [Fasciola hepatica]
38	2387	CAO79608.1	33,84	3	3	0	1	1	1		49632	beta-tubulin [Fasciola hepatica]
38	2388	THD26917.1	33,84	3	3	0	1	1	1		49604	Tubulin beta chain [Fasciola hepatica]
38	2389	CAP72050.1	33,84	3	3	0	1	1	1		49604	tubulin beta-2 [Fasciola hepatica]
38	2390	CAO79609.1	33,84	3	3	0	1	1	1		49849	beta-tubulin [Fasciola hepatica]
38	2391	THD24105.1	33,84	3	3	0	1	1	1		49819	Tubulin beta chain [Fasciola hepatica]
38	2392	THD27086.1	33,84	3	3	0	1	1	1		49819	Tubulin beta chain [Fasciola hepatica]
38	2393	CAP72051.1	33,84	3	3	0	1	1	1		49819	tubulin beta-3 [Fasciola hepatica]
38	2394	THD22032.1	33,84	3	3	0	1	1	1		49861	Tubulin beta chain [Fasciola hepatica]
38	2395	CAP72049.1	33,84	3	3	0	1	1	1		49861	tubulin beta-1 [Fasciola hepatica]
38	2396	CAO79607.1	33,84	3	3	0	1	1	1		49861	beta-tubulin [Fasciola hepatica]
35	180	THD25594.1	33,25	0	0	0	1	1	1		284868	Filamin-A [Fasciola hepatica]
36	47	THD25068.1	31,03	0	0	0	1	1	1		269396	Filamin-A [Fasciola hepatica]
44	140	THD17940.1	30,26	12	12	0	1	1	1		10871	Titin partial [Fasciola hepatica]
39	6240	THD26833.1	28,11	1	1	0	1	1	1	Dehydration	97102	Intraflagellar transport protein 88 [Fasciola hepatica]
26	431	THD27070.1	28,07	2	2	0	1	1	2		53021	Dihydrolipoamide acetyltransferase component of pyruvate dehydrogenase complex [Fasciola hepatica]
46	6241	THD25691.1	27,79	2	2	0	1	1	1		63206	Fibrillin-2 [Fasciola hepatica]
47	1810	THD26946.1	26,96	5	5	0	1	1	1	Carbamidomethylation; Oxidation (M); Dehydration	74265	hypothetical protein D915_002207 [Fasciola hepatica]

FIGURE 7, LANE 3A

Protein Group	Protein ID	Accession	-10lgP	Coverage (%)	Coverage (%) 4	Area 4	#Peptides	#Unique	#Spec 4	PTM	Avg. Mass	Description
5	16	ADP09371.1	160,39	29	29		15	0	58	Deamidation (NQ); Oxidation (M); Dehydration	36663	cathepsin L-like proteinase [Fasciola hepatica]
2	17	AAM11647.1	158	27	27		13	0	59	Carbamidomethylation; Deamidation (NQ); Oxidation (M); Dehydration	35190	cathepsin L partial [Fasciola hepatica]
2	19	AAA29136.1	158	26	26		13	0	59	Carbamidomethylation; Deamidation (NQ); Oxidation (M); Dehydration	36896	cathepsin [Fasciola hepatica]
2	20	sp|Q24940.1|CATLL_FASHE	158	26	26		13	0	59	Carbamidomethylation; Deamidation (NQ); Oxidation (M); Dehydration	36896	RecName: Full=Cathepsin L-like proteinase; Flags: Precursor
3	28	pdb|2O6X|A	157,89	29	29		13	0	59	Carbamidomethylation; Deamidation (NQ); Oxidation (M); Dehydration	35070	Chain A Secreted cathepsin L 1
3	29	AAB41670.2	157,89	28	28		13	0	59	Carbamidomethylation; Deamidation (NQ); Oxidation (M); Dehydration	36773	secreted cathepsin L 1 [Fasciola hepatica]
1	9	AAR99518.1	155,57	22	22		13	0	61	Deamidation (NQ); Oxidation (M); Dehydration	36714	cathepsin L protein [Fasciola hepatica]
4	4	CAC12806.1	150,68	26	26		13	0	58	Deamidation (NQ); Oxidation (M)	35196	cathepsin L1 partial [Fasciola hepatica]
6	21	AAK38169.1	146,02	23	23		12	0	45	Oxidation (M); Dehydration	35234	cathepsin L-like partial [Fasciola hepatica]
10	23	ACJ12894.1	145,59	29	29	0	12	3	39	Carbamidomethylation; Oxidation (M)	36557	cathepsin L1D [Fasciola hepatica]
10	22	ACJ12893.1	145,59	29	29	0	12	3	39	Carbamidomethylation; Oxidation (M)	36548	cathepsin L1D [Fasciola hepatica]
7	39	BAA23743.1	142,53	20	20	0	11	1	44	Oxidation (M); Dehydration	36726	cathepsin L [Fasciola hepatica]
9	6	ATW63990.1	129,5	16	16	2,67E+03	6	1	40	Carbamidomethylation; Deamidation (NQ)	35466	procathepsin L5 partial [Fasciola hepatica]
9	7	AAA29137.1	129,5	16	16	2,67E+03	6	1	40	Carbamidomethylation; Deamidation (NQ)	37177	cathepsin [Fasciola hepatica]
9	8	AAF76330.1	129,5	16	16	2,67E+03	6	1	40	Carbamidomethylation; Deamidation (NQ)	37149	cathepsin L [Fasciola hepatica]
8	38	AAR99519.1	129,48	28	28		11	0	41	Oxidation (M)	26217	cathepsin L protein [Fasciola hepatica]
12	50	THD18939.1	101,92	17	17	0	4	1	20	Oxidation (M)	26416	Secreted cathepsin L 1 [Fasciola hepatica]
16	58	ABG00259.1	95,08	21	21		5	0	8		24555	cathepsin L2 partial [Fasciola hepatica]
15	32	ASK40163.1	94,44	14	14		5	0	9		35438	L2 procathepsin partial [Fasciola hepatica]
15	33	AAC47721.1	94,44	13	13		5	0	9		37033	secreted cathepsin L 2 [Fasciola hepatica]
15	34	ABN50361.2	94,44	13	13		5	0	9		36981	cathepsin L [Fasciola hepatica]
15	35	ABQ95351.1	94,44	13	13		5	0	9		37072	secreted cathepsin L2 [Fasciola hepatica]
15	40	CAA80446.1	94,44	13	13		5	0	9		37159	cathepsin L-like protease [Fasciola hepatica]
11	59	THD18417.1	94,36	18	18	1,67E+03	4	1	25	Acetylation (Protein N-term); Deamidation (NQ)	14678	Cathepsin L [Fasciola hepatica]
18	13	pdb|2VYW|A	86,9	24	24	0	4	4	6		16560	Chain A HEMOGLOBIN
18	14	THD25867.1	86,9	24	24	0	4	4	6		16691	Myoglobin 2 [Fasciola hepatica]
18	15	ABW96608.1	86,9	24	24	0	4	4	6		16691	hemoglobin F2 [Fasciola hepatica]
20	2473	pdb|2WB9|A	79,19	16	16	1,17E+03	3	3	4	Oxidation (M)	24534	Chain A Glutathione Transferase Sigma Class
20	2475	pdb|2WDU|A	79,19	16	16	1,17E+03	3	3	4	Oxidation (M)	24534	Chain A GLUTATHIONE TRANSFERASE SIGMA CLASS
20	2477	pdb|2WDU|B	79,19	16	16	1,17E+03	3	3	4	Oxidation (M)	24534	Chain B GLUTATHIONE TRANSFERASE SIGMA CLASS
20	2478	pdb|2WB9|B	79,19	16	16	1,17E+03	3	3	4	Oxidation (M)	24534	Chain B Glutathione Transferase Sigma Class
20	2474	THD27894.1	79,19	16	16	1,17E+03	3	3	4	Oxidation (M)	24610	Prostaglandin-H2 D-isomerase [Fasciola hepatica]
20	2476	ABI79450.1	79,19	16	16	1,17E+03	3	3	4	Oxidation (M)	24534	glutathione transferase sigma class [Fasciola hepatica]
13	60	THD23701.1	72,03	5	5		2	0	19		37063	Secreted cathepsin L 1 [Fasciola hepatica]
22	130	THD18112.1	50,17	8	8	3,19E+03	1	1	4		13016	Cathepsin L protease [Fasciola hepatica]
35	9059	CAA06158.1	38,74	4	4	0	1	1	1		21748	thiol-specific antioxidant protein [Fasciola hepatica]
35	9060	AAB71727.1	38,74	4	4	0	1	1	1		21661	peroxiredoxin [Fasciola hepatica]
35	9061	sp|P91883.1|TDX_FASHE	38,74	4	4	0	1	1	1		21661	RecName: Full=Thioredoxin peroxidase; AltName: Full=Peroxiredoxin; AltName: Full=Thio
35	9062	ACI04165.1	38,74	4	4	0	1	1	1		24577	thioredoxin peroxidase [Fasciola hepatica]
35	9063	THD25297.1	38,74	3	3	0	1	1	1		27280	Thioredoxin peroxidase [Fasciola hepatica]
31	47	THD25068.1	37,91	0	0	0	1	1	1		269396	Filamin-A [Fasciola hepatica]
30	182	THD19813.1	37,47	17	17	5,36E+03	1	1	2	Carbamidomethylation; Deamidation (NQ)	8669	Cathepsin L [Fasciola hepatica]
25	3342	THD25022.1	35,23	3	3	0	1	1	3		30591	hypothetical protein D915_003906 [Fasciola hepatica]
33	9054	pdb|2FHE|A	34,94	4	4	0	1	1	1		25119	Chain A Glutathione S-transferase
33	9055	pdb|2FHE|B	34,94	4	4	0	1	1	1		25119	Chain B Glutathione S-transferase
33	9056	prf||1905266D	34,94	4	4	0	1	1	1		25730	glutathione S transferase:ISOTYPE=GST1
33	9057	sp|P56598.2|GST29_FASHE	34,94	4	4	0	1	1	1		25730	RecName: Full=Glutathione S-transferase class-mu 26 kDa isozyme 1; Short=GST1; AltNan
33	9064	CAA00118.1	34,94	4	4	0	1	1	1		23073	glutathione-S-transferase partial [Fasciola hepatica]
33	9065	THD21358.1	34,94	3	3	0	1	1	1		27236	Mu-class gst glutathione S-transferase [Fasciola hepatica]
32	1	THD20935.1	34,21	5	5	0	1	1	1		14635	Fatty acid binding protein a [Fasciola hepatica]
32	2	sp|Q9U1G6.1|FABP3_FASHE	34,21	5	5	0	1	1	1		14623	RecName: Full=Fatty acid-binding protein type 3
32	3	CAB65015.1	34,21	5	5	0	1	1	1		14623	fatty acid binding protein [Fasciola hepatica]
36	9066	THD28418.1	33,39	4	4	0	1	1	1		20984	hypothetical protein D915_000689 [Fasciola hepatica]
26	197	ABZ80402.1	32,93	3	3	6,38E+03	1	1	2	Dehydration	34581	cathepsin L6 partial [Fasciola hepatica]
26	2588	ABF85681.1	32,93	4	4	6,38E+03	1	1	2	Dehydration	26007	cathepsin L3 partial [Fasciola hepatica]
26	2589	CAC12807.1	32,93	3	3	6,38E+03	1	1	2	Dehydration	35174	procathepsin L3 partial [Fasciola hepatica]
26	1253	ABW75768.2	32,93	3	3	6,38E+03	1	1	2	Dehydration	35718	procathepsin L partial [Fasciola hepatica]
26	1255	ACM67633.1	32,93	3	3	6,38E+03	1	1	2	Dehydration	37216	cathepsin 1L [Fasciola hepatica]
26	1256	QPX50259.1	32,93	3	3	6,38E+03	1	1	2	Dehydration	37306	cathepsin L3 peptidase [Fasciola hepatica]
26	123	ABZ80399.1	32,93	3	3	6,38E+03	1	1	2	Dehydration	35333	cathepsin L3 partial [Fasciola hepatica]
26	1254	ABW24657.1	32,93	3	3	6,38E+03	1	1	2	Dehydration	37418	cathepsin L [Fasciola hepatica]
26	125	ACM67632.1	32,93	3	3	6,38E+03	1	1	2	Dehydration	37455	cathepsin 2L [Fasciola hepatica]
28	141	THD19552.1	31,93	3	3	0	1	1	2	Dehydration	36643	Cysteine protease [Fasciola hepatica]
28	2491	ABZ80398.1	31,93	3	3	0	1	1	2	Dehydration	35151	cathepsin L3 partial [Fasciola hepatica]
28	2492	CAC12805.1	31,93	3	3	0	1	1	2	Dehydration	35409	procathepsin L3 partial [Fasciola hepatica]
28	2493	CAM57967.1	31,93	3	3	0	1	1	2	Dehydration	36012	unnamed protein product partial [Fasciola hepatica]
28	9067	sp|P80532.1|CATL3_FASHE	31,93	47	47	0	1	1	2	Dehydration	2242	RecName: Full=Putative cathepsin L3; AltName: Full=Newly excysted juvenile protein 8

28	9068	AAB35021.1	31,93	47	47	0	1	1	2	Dehydration		2242	somatic protein 8=cathepsin L homolog {N-terminal} [Fasciola hepatica newly excysted ju
27	121	THD27507.1	31,19	3	3	0	1	1	2			27325	Glutathione S-transferase omega class [Fasciola hepatica]
27	122	AFX98104.1	31,19	3	3	0	1	1	2			27334	glutathione S-transferase omega class [Fasciola hepatica]
37	4009	ABX58011.1	30,07	27	27	0	1	1	1	Carbamidomethylation; Dehydration		18577	pumilio-like protein 2 partial [Fasciola hepatica]
37	4010	ABX58012.1	30,07	27	27	0	1	1	1	Carbamidomethylation; Dehydration		18536	pumilio-like protein 2 partial [Fasciola hepatica]
37	2723	THD27975.1	30,07	4	4	0	1	1	1	Carbamidomethylation; Dehydration		124244	Maternal protein pumilio [Fasciola hepatica]
34	45	THD25752.1	29,44	10	10	0	1	1	1	Oxidation (M)		14712	Fatty acid-binding protein type 2 [Fasciola hepatica]
34	46	sp|Q7M4G0.3|FABP1_FASHE	29,44	10	10	0	1	1	1	Oxidation (M)		14712	RecName: Full=Fatty acid-binding protein Fh15
612	215	THD26995.1	28,05	0	0		0	0	0		1023	161671	RNA polymerase II subunit A C-terminal domain phosphatase [Fasciola hepatica]

FIGURE 7, LANE 3B

Protein Group	Protein ID	Accession	-10lgP	Coverage (%)	Coverage (%) 5	Area 5	#Peptides	#Unique	#Spec 5	PTM	Avg. Mass	Description
1	1	THD20935.1	174,66	60	60	4,72E+05	15	15	133	Carbamidomethylation; Deami	14635	Fatty acid binding protein a [Fasciola hepatica]
1	2	sp|Q9U1G6.1|FABP3_FASHE	174,66	60	60	4,72E+05	15	15	133	Carbamidomethylation; Deami	14623	RecName: Full=Fatty acid-binding protein type 3
1	3	CAB65015.1	174,66	60	60	4,72E+05	15	15	133	Carbamidomethylation; Deami	14623	fatty acid binding protein [Fasciola hepatica]
3	13	pdb|2VYW|A	165,38	40	40	5,35E+03	12	2	107	Deamidation (NQ); Dehydratior	16560	Chain A HEMOGLOBIN
3	14	THD25867.1	165,38	40	40	5,35E+03	12	2	107	Deamidation (NQ); Dehydratior	16691	Myoglobin 2 [Fasciola hepatica]
3	15	ABW96608.1	165,38	40	40	5,35E+03	12	2	107	Deamidation (NQ); Dehydratior	16691	hemoglobin F2 [Fasciola hepatica]
2	12	THD25868.1	164,18	40	40	3,79E+04	12	2	113	Deamidation (NQ); Dehydratior	16722	Myoglobin 2 [Fasciola hepatica]
4	45	THD25752.1	154,4	45	45	1,83E+05	11	10	75	Carbamidomethylation; Acetyla	14712	Fatty acid-binding protein type 2 [Fasciola hepatica]
4	46	sp|Q7M4G0.3|FABP1_FASHE	154,4	45	45	1,83E+05	11	10	75	Carbamidomethylation; Acetyla	14712	RecName: Full=Fatty acid-binding protein Fh15
5	10	THD25753.1	136,23	52	52	1,62E+05	10	9	52	Acetylation (Protein N-term); D	14936	Fatty acid-binding protein type V [Fasciola hepatica]
5	11	sp|Q7M4G1.2|FABP2_FASHE	136,23	52	52	1,62E+05	10	9	52	Acetylation (Protein N-term); D	14936	RecName: Full=Fatty acid-binding protein type 2
6	26	THD25871.1	135,82	34	34	1,12E+04	8	5	28	Oxidation (M)	17084	Globin-3 [Fasciola hepatica]
10	30	THD25869.1	109,84	33	33	2,96E+04	4	3	16	Deamidation (NQ)	14232	Myoglobin 2 [Fasciola hepatica]
7	4	CAC12806.1	94,62	10	10	1,69E+04	3	1	27	Oxidation (M)	35196	cathepsin L1 partial [Fasciola hepatica]
9	9	AAR99518.1	93,37	9	9	2,87E+04	3	1	19	Oxidation (M)	36714	cathepsin L protein [Fasciola hepatica]
12	31	THD25273.1	87,08	3	3	0	5	5	7		171980	Multi-domain cystatin [Fasciola hepatica]
15	36	CAB65014.1	86,82	30	30	2,92E+03	3	3	6		11683	thioredoxin (TRX) [Fasciola hepatica]
15	37	pdb|2VIM|A	86,82	30	30	2,92E+03	3	3	6		11683	Chain A Thioredoxin
11	68	THD25870.1	84,52	30	30	5,10E+03	4	2	14	Oxidation (M)	8022	hypothetical protein D915_003343 [Fasciola hepatica]
13	73	THD18299.1	78,18	31	31	0	4	4	7	Oxidation (M)	12063	Mitochondrial cytochrome c [Fasciola hepatica]
17	85	THD21681.1	75,51	23	23	8,13E+02	3	3	5		17348	Calmodulin/ calcium-binding protein/ EF-Hand superfamily pro
17	86	CAL91033.1	75,51	23	23	8,13E+02	3	3	5		17348	calmodulin-like protein 2 (CaM2) [Fasciola hepatica]
14	395	THD26047.1	74,07	25	25	6,03E+02	3	3	6	Deamidation (NQ); Oxidation (I	15372	Fatty acid-binding protein type V [Fasciola hepatica]
14	396	AJO53793.1	74,07	25	25	6,03E+02	3	3	6	Deamidation (NQ); Oxidation (I	15372	fatty acid-binding protein type V [Fasciola hepatica]
31	54	AAD30361.1	67,95	16	16	0	2	2	2	Oxidation (M)	14979	Cu/Zn-superoxide dismutase [Fasciola hepatica]
31	53	THD25954.1	67,95	15	15	0	2	2	2	Oxidation (M)	15826	Superoxide dismutase [Fasciola hepatica]
19	75	THD22756.1	67,59	9	9	2,20E+03	2	2	5		24823	hypothetical protein D915_006522 [Fasciola hepatica]
27	3120	THD24741.1	66,63	20	20	0	3	3	3		15708	Eukaryotic translation initiation factor 3 subunit A [Fasciola hep
21	3121	THD23037.1	63,76	1	1	0	2	2	4		269992	Spectrin beta chain [Fasciola hepatica]
20	58	ABG00259.1	63,48	13	13	0	3	3	4		24555	cathepsin L2 partial [Fasciola hepatica]
20	32	ASK40163.1	63,48	9	9	0	3	3	4		35438	L2 procathepsin partial [Fasciola hepatica]
20	33	AAC47721.1	63,48	9	9	0	3	3	4		37033	secreted cathepsin L 2 [Fasciola hepatica]
20	35	ABQ95351.1	63,48	9	9	0	3	3	4		37072	secreted cathepsin L2 [Fasciola hepatica]
20	40	CAA80446.1	63,48	9	9	0	3	3	4		37159	cathepsin L-like protease [Fasciola hepatica]
20	34	ABN50361.2	63,48	9	9	0	3	3	4		36981	cathepsin L [Fasciola hepatica]
29	3122	THD20946.1	58,36	7	7	0	3	3	3	Oxidation (M)	25264	hypothetical protein D915_008270 [Fasciola hepatica]
25	84	THD24007.1	56,5	12	12	2,52E+03	2	2	3		17889	Myophilin [Fasciola hepatica]
23	131	THD19897.1	52,63	10	10	0	1	1	4		11069	Stefin-1 [Fasciola hepatica]
34	3123	THD21074.1	48,68	3	3	0	1	1	2	Deamidation (NQ)	43374	Severin [Fasciola hepatica]
24	129	THD19782.1	48,38	5	5	2,00E+03	1	1	4		18531	hypothetical protein D915_009347 [Fasciola hepatica]
35	130	THD18112.1	46,11	8	8	0	1	1	2		13016	Cathepsin L protease [Fasciola hepatica]
28	127	AIE76459.1	45,57	8	8	0	1	1	3		14077	CD59-like protein [Fasciola hepatica]
28	128	THD28115.1	45,57	8	8	0	1	1	3		14077	CD59 protein [Fasciola hepatica]
36	87	THD23439.1	43,77	8	8	0	1	1	2	Oxidation (M)	17732	Peptidyl-prolyl cis-trans isomerase [Fasciola hepatica]
33	47	THD25068.1	41,58	1	1	0	2	2	2		269396	Filamin-A [Fasciola hepatica]
26	3124	THD22889.1	36,97	10	10	0	1	1	3	Oxidation (M)	10822	Glycine cleavage system protein H [Fasciola hepatica]
37	140	THD17940.1	35,3	12	12	0	1	1	2		10871	Titin partial [Fasciola hepatica]
38	151	THD24822.1	32,25	1	1	1,02E+03	1	1	2	Deamidation (NQ)	92519	Sodium and chloride dependent glycine [Fasciola hepatica]
45	3127	THD25087.1	30,32	4	4	0	1	1	1		17699	Ubiquitin conjugating enzyme [Fasciola hepatica]
43	3128	THD22837.1	29,39	5	5	0	1	1	1		16642	Ubiquitin-conjugating enzyme [Fasciola hepatica]
32	3129	AFM84631.1	29,03	5	5	0	1	1	2		17041	CaM3 [Fasciola hepatica]

32	3130	THD20804.1	29,03	5	5	0	1	1	2	17041	CaM3 [Fasciola hepatica]
40	64	THD25658.1	27,71	1	1	0	1	1	1	73844	L-plastin [Fasciola hepatica]
39	206	THD29031.1	26,89	1	1	0	1	1	2	94546	Basement membrane-specific heparan sulfate proteoglycan cor
46	3131	THD18631.1	26,58	4	4	0	1	1	1	22198	Prostaglandin e synthase [Fasciola hepatica]
42	200	THD27851.1	25,28	1	1	0	1	1	1	82596	T-cell immunomodulatory protein [Fasciola hepatica]
41	3132	THD21625.1	24,58	1	1	0	1	1	1	79261	hypothetical protein D915_007782 [Fasciola hepatica]
44	181	THD28589.1	24,44	1	1	0	1	1	1	190619	Tensin [Fasciola hepatica]
47	204	THD26611.1	23,85	1	1	0	1	1	1	110171	Titin [Fasciola hepatica]
48	3134	THD28530.1	23,52	2	2	0	1	1	1	47221	Dihydrolipoamide acetyltransferase component of pyruvate de
49	209	THD21260.1	23,36	1	1	0	1	1	1	80464	von Willebrand factor A domain-containing protein 5A [Fasciola

FIGURE 7, LANE 3C

Protein Group	Protein ID	Accession	-10lgP	Coverage (%)	Coverage (%) 6	Area 6	#Peptides	#Unique	#Spec 6	PTM	Avg. Mass	Description
3	45	THD25752.1	143,84	39	39	7,32E+04	8	8	45	Carbamidomethylation; Oxidation (M)	14712	Fatty acid-binding protein type 2 [Fasciola hepatica]
3	46	sp|Q7M4G0.3|FABP1_FASHE	143,84	39	39	7,32E+04	8	8	45	Carbamidomethylation; Oxidation (M)	14712	RecName: Full=Fatty acid-binding protein Fh15
2	13	pdb|2VYW|A	139,2	40	40	4,67E+02	9	2	48	Deamidation (NQ); Dehydration	16560	Chain A HEMOGLOBIN
2	14	THD25867.1	139,2	40	40	4,67E+02	9	2	48	Deamidation (NQ); Dehydration	16691	Myoglobin 2 [Fasciola hepatica]
2	15	ABW96608.1	139,2	40	40	4,67E+02	9	2	48	Deamidation (NQ); Dehydration	16691	hemoglobin F2 [Fasciola hepatica]
4	11	sp|Q7M4G1.2|FABP2_FASHE	138,81	49	49	3,80E+04	12	12	44	Oxidation (M)	14936	RecName: Full=Fatty acid-binding protein type 2
4	10	THD25753.1	138,81	49	49	3,80E+04	12	12	44	Oxidation (M)	14936	Fatty acid-binding protein type V [Fasciola hepatica]
6	1	THD20935.1	138,14	45	45	5,17E+04	10	10	41	Deamidation (NQ); Oxidation (M); Dehydration	14635	Fatty acid binding protein a [Fasciola hepatica]
6	2	sp|Q9U1G6.1|FABP3_FASHE	138,14	45	45	5,17E+04	10	10	41	Deamidation (NQ); Oxidation (M); Dehydration	14623	RecName: Full=Fatty acid-binding protein type 3
6	3	CAB65015.1	138,14	45	45	5,17E+04	10	10	41	Deamidation (NQ); Oxidation (M); Dehydration	14623	fatty acid binding protein [Fasciola hepatica]
1	12	THD25868.1	137,96	40	40	1,43E+04	9	2	51	Deamidation (NQ); Dehydration	16722	Myoglobin 2 [Fasciola hepatica]
5	36	CAB65014.1	134,62	61	61	3,44E+04	9	9	42	Deamidation (NQ); Oxidation (M)	11683	thioredoxin (TRX) [Fasciola hepatica]
5	37	pdb|2VIM|A	134,62	61	61	3,44E+04	9	9	42	Deamidation (NQ); Oxidation (M)	11683	Chain A Thioredoxin
7	4	CAC12806.1	117,17	17	17		7	0	22	Oxidation (M)	35196	cathepsin L1 partial [Fasciola hepatica]
13	6	ATW63990.1	114,47	15	15	0	5	1	15	Carbamidomethylation; Oxidation (M)	35466	procathepsin L5 partial [Fasciola hepatica]
13	7	AAA29137.1	114,47	14	14	0	5	1	15	Carbamidomethylation; Oxidation (M)	37177	cathepsin [Fasciola hepatica]
13	8	AAF76330.1	114,47	14	14	0	5	1	15	Carbamidomethylation; Oxidation (M)	37149	cathepsin L [Fasciola hepatica]
15	30	THD25869.1	110,1	48	48	3,99E+03	6	4	13	Deamidation (NQ)	14232	Myoglobin 2 [Fasciola hepatica]
16	26	THD25871.1	108,87	33	33	0	7	5	8		17084	Globin-3 [Fasciola hepatica]
8	9	AAR99518.1	107,4	15	15		6	0	18	Oxidation (M)	36714	cathepsin L protein [Fasciola hepatica]
10	22	ACJ12893.1	100,07	14	14	0	5	1	17	Oxidation (M)	36548	cathepsin L1D [Fasciola hepatica]
10	23	ACJ12894.1	100,07	14	14	0	5	1	17	Oxidation (M)	36557	cathepsin L1D [Fasciola hepatica]
10	24	QIB89441.1	100,07	7	7	0	5	1	17	Oxidation (M)	69865	cathepsin L7 [Fasciola hepatica]
11	131	THD19897.1	99,78	21	21	7,22E+03	4	4	17		11069	Stefin-1 [Fasciola hepatica]
12	17	AAM11647.1	98,91	17	17	0	6	1	15	Carbamidomethylation; Oxidation (M)	35190	cathepsin L partial [Fasciola hepatica]
12	19	AAA29136.1	98,91	17	17	0	6	1	15	Carbamidomethylation; Oxidation (M)	36896	cathepsin [Fasciola hepatica]
12	20	sp|Q24940.1|CATLL_FASHE	98,91	17	17	0	6	1	15	Carbamidomethylation; Oxidation (M)	36896	RecName: Full=Cathepsin L-like proteinase; Flags: Precursor
14	6226	THD25975.1	96,55	28	28	3,43E+03	7	4	13	Oxidation (M)	22035	Tegumental calcium-binding EF-hand protein 4 [Fasciola hepatica]
14	6227	CAA06036.1	96,55	28	28	3,43E+03	7	4	13	Oxidation (M)	22028	calcium-binding protein [Fasciola hepatica]
14	6228	AML33332.1	96,55	28	28	3,43E+03	7	4	13	Oxidation (M)	22035	CaBP1 [Fasciola hepatica]
19	59	THD18417.1	85,23	23	23	0	4	1	7	Oxidation (M)	14678	Cathepsin L [Fasciola hepatica]
17	6231	THD25974.1	83,91	16	16	0	5	2	8	Oxidation (M)	22259	Tegumental calcium-binding EF-hand protein 4 [Fasciola hepatica]
17	6232	CAA06035.1	83,91	16	16	0	5	2	8	Oxidation (M)	22259	calcium-binding protein [Fasciola hepatica]
17	6233	AJF23779.1	83,91	16	16	0	5	2	8	Oxidation (M)	22233	calcium binding protein [Fasciola hepatica]
20	11954	THD28602.1	83,6	51	51	0	5	5	7		7666	Calcium binding protein [Fasciola hepatica]
20	11955	AAF31420.1	83,6	51	51	0	5	5	7		7666	putative calcium-binding protein [Fasciola hepatica]
28	6224	THD19920.1	80,95	20	20	0	4	4	4		17188	Polyubiquitin-B [Fasciola hepatica]
28	6225	THD18230.1	80,95	8	8	0	4	4	4		42830	Polyubiquitin-C [Fasciola hepatica]
18	78	THD19412.1	76,64	33	33	9,03E+02	4	4	8	Oxidation (M)	12490	Type-1 cystatin cysteine protease inhibitor [Fasciola hepatica]
18	79	AAV68752.1	76,64	33	33	9,03E+02	4	4	8	Oxidation (M)	12472	cystatin [Fasciola hepatica]
22	11956	AFM84632.1	74,81	10	10	0	3	3	6		22250	CaBP4 [Fasciola hepatica]
26	73	THD18299.1	73,53	24	24	0	3	3	5	Oxidation (M)	12063	Mitochondrial cytochrome c [Fasciola hepatica]
35	87	THD23439.1	68,55	9	9	0	2	2	2	Oxidation (M)	17732	Peptidyl-prolyl cis-trans isomerase [Fasciola hepatica]
33	57	THD28453.1	68,34	3	3	0	2	2	2		83852	Titin [Fasciola hepatica]
27	84	THD24007.1	60,09	7	7	5,65E+02	2	2	5		17889	Myophilin [Fasciola hepatica]
30	58	ABG00259.1	59,53	11	11	0	3	1	3	Oxidation (M)	24555	cathepsin L2 partial [Fasciola hepatica]
36	3124	THD22889.1	54,75	20	20	0	2	2	2	Oxidation (M)	10822	Glycine cleavage system protein H [Fasciola hepatica]
32	68	THD25870.1	53,16	27	27	0	3	2	3		8022	hypothetical protein D915_003343 [Fasciola hepatica]
37	209	THD21260.1	47,12	1	1	0	2	2	2		80464	von Willebrand factor A domain-containing protein 5A [Fasciola hepatic
38	3120	THD24741.1	46,35	12	12	0	2	2	2		15708	Eukaryotic translation initiation factor 3 subunit A [Fasciola hepatica]
42	3139	THD18728.1	46,15	7	7	0	1	1	2		16439	hypothetical protein D915_010606 [Fasciola hepatica]
43	11958	THD21472.1	45,45	9	9	0	1	1	2		11135	Dynein light chain 1 cytoplasmic [Fasciola hepatica]
44	6234	THD23562.1	44,12	2	2	0	1	1	2		57624	Ubiquilin [Fasciola hepatica]
52	6237	THD28916.1	43,6	12	12	0	1	1	1	Oxidation (M)	9818	hypothetical protein D915_000254 [Fasciola hepatica]
53	140	THD17940.1	41,61	12	12	0	1	1	1		10871	Titin partial [Fasciola hepatica]
39	6236	THD24513.1	40,23	2	2	1,54E+02	1	1	2	Oxidation (M)	78182	Enamine/imine deaminase [Fasciola hepatica]
49	7238	THD19385.1	39,91	4	4	0	1	1	1		28984	Phosphoglycerate mutase [Fasciola hepatica]
29	127	AIE76459.1	39,8	8	8	6,60E+02	1	1	4		14077	CD59-like protein [Fasciola hepatica]

29	128	THD28115.1	39,8	8	8	6,60E+02	1	1	4			14077	CD59 protein [Fasciola hepatica]
34	3861	THD19187.1	37,11	2	2	0	1	1	3			47299	Dihydrolipoyllysine-residue succinyltransferase component of 2-oxoglu
23	177	CEL12048.1	36,9	8	8	6,17E+03	1	1	5			9252	Kunitz [Fasciola hepatica]
23	176	THD28325.1	36,9	8	8	6,17E+03	1	1	5			9314	Kunitz-CH [Fasciola hepatica]
23	175	AAB46830.1	36,9	12	12	6,17E+03	1	1	5			6589	Fh-KTM=6.751 kda monomeric Kunitz-type proteinase inhibitor [Fasciol
23	178	CEL12049.1	36,9	8	8	6,17E+03	1	1	5			9356	Kunitz-CH [Fasciola hepatica]
54	130	THD18112.1	36,79	8	8	0	1	1	1			13016	Cathepsin L protease [Fasciola hepatica]
55	182	THD19813.1	36,41	17	17	0	1	1	1	Carbamidomethylation; Deamidation (NQ)		8669	Cathepsin L [Fasciola hepatica]
47	3123	THD21074.1	35,79	2	2	0	1	1	1			43374	Severin [Fasciola hepatica]
40	11959	THD18409.1	35,37	8	8	0	1	1	2			10501	hypothetical protein D915_011126 [Fasciola hepatica]
45	11960	THD24909.1	34,32	6	6	0	1	1	2			17996	DnaJ subfamily B member 4 [Fasciola hepatica]
50	64	THD25658.1	33,74	1	1	0	1	1	1			73844	L-plastin [Fasciola hepatica]
51	11961	THD24394.1	32,56	11	11	0	1	1	1			8480	hypothetical protein D915_004833 [Fasciola hepatica]
46	206	THD29031.1	32,18	1	1	0	1	1	1			94546	Basement membrane-specific heparan sulfate proteoglycan core protei
56	11962	THD26015.1	30,9	3	3	0	1	1	1			24478	Thioredoxin dependent peroxide reductase [Fasciola hepatica]
58	431	THD27070.1	28,68	2	2	0	1	1	1			53021	Dihydrolipoamide acetyltransferase component of pyruvate dehydroge
48	47	THD25068.1	28,41	0	0	0	1	1	1			269396	Filamin-A [Fasciola hepatica]
59	6288	THD26426.1	27,86	9	9	8,80E+02	1	1	1	Deamidation (NQ)		9042	hypothetical protein D915_002838 [Fasciola hepatica]
41	200	THD27851.1	26,56	1	1	1,91E+03	1	1	2		997	82596	T-cell immunomodulatory protein [Fasciola hepatica]

FIGURE 7, LANE 4A

Protein Group	Protein ID	Accession	-10lgP	Coverage (%)	Coverage (%)	Area 7	#Peptides	#Unique	#Spec 7	PTM	Avg. Mass	Description
5	28	pdb|2O6X|A	140,63	18	18		8	0	38	Carbamidome	35070	Chain A Secreted cathepsin L 1
5	29	AAB41670.2	140,63	17	17		8	0	38	Carbamidome	36773	secreted cathepsin L 1 [Fasciola hepatica]
5	27	AAP49831.1	137,24	17	17		8	0	38	Carbamidome	36602	cathepsin L partial [Fasciola hepatica]
4	16	ADP09371.1	139,74	19	19		9	0	39	Dehydration	36663	cathepsin L-like proteinase [Fasciola hepatica]
3	19	AAA29136.1	139,66	16	16		8	0	39	Carbamidome	36896	cathepsin [Fasciola hepatica]
3	20	sp|Q24940.1|	139,66	16	16		8	0	39	Carbamidome	36896	RecName: Full=Cathepsin L-like proteinase; Flags: Precursor
3	17	AAM11647.1	139,66	17	17		8	0	39	Carbamidome	35190	cathepsin L partial [Fasciola hepatica]
2	4	CAC12806.1	138,11	20	20		9	0	48	Deamidation (	35196	cathepsin L1 partial [Fasciola hepatica]
1	9	AAR99518.1	136,32	15	15		8	0	52	Deamidation (	36714	cathepsin L protein [Fasciola hepatica]
8	23	ACJ12894.1	134,05	26	26	0	10	2	34	Carbamidome	36557	cathepsin L1D [Fasciola hepatica]
8	22	ACJ12893.1	134,05	26	26	0	10	2	34	Carbamidome	36548	cathepsin L1D [Fasciola hepatica]
6	6	ATW63990.1	124,13	16	16	0	5	1	38	Carbamidome	35466	procathepsin L5 partial [Fasciola hepatica]
6	7	AAA29137.1	124,13	16	16	0	5	1	38	Carbamidome	37177	cathepsin [Fasciola hepatica]
6	8	AAF76330.1	124,13	16	16	0	5	1	38	Carbamidome	37149	cathepsin L [Fasciola hepatica]
7	21	AAK38169.1	120,96	16	16		8	0	37	Dehydration	35234	cathepsin L-like partial [Fasciola hepatica]
9	38	AAR99519.1	114,57	21	21		8	0	32		26217	cathepsin L protein [Fasciola hepatica]
10	59	THD18417.1	90,28	18	18	0	3	1	21	Deamidation (	14678	Cathepsin L [Fasciola hepatica]
16	58	ABG00259.1	87,01	21	21		5	0	9		24555	cathepsin L2 partial [Fasciola hepatica]
15	32	ASK40163.1	84,89	14	14		5	0	10		35438	L2 procathepsin partial [Fasciola hepatica]
15	33	AAC47721.1	84,89	13	13		5	0	10		37033	secreted cathepsin L 2 [Fasciola hepatica]
15	34	ABN50361.2	84,89	13	13		5	0	10		36981	cathepsin L [Fasciola hepatica]
15	35	ABQ95351.1	84,89	13	13		5	0	10		37072	secreted cathepsin L2 [Fasciola hepatica]
15	40	CAA80446.1	84,89	13	13		5	0	10		37159	cathepsin L-like protease [Fasciola hepatica]
12	60	THD23701.1	70,12	5	5		2	0	17		37063	Secreted cathepsin L 1 [Fasciola hepatica]
20	130	THD18112.1	39,8	8	8	0	1	1	1		13016	Cathepsin L protease [Fasciola hepatica]
21	15008	THD26068.1	32,73	1	1	0	1	1	1	Deamidation (	91604	Transcription factor glial cells missing [Fasciola hepatica]
19	197	ABZ80402.1	31,73	3	3	1,13E+03	1	1	1	Dehydration	34581	cathepsin L6 partial [Fasciola hepatica]
19	2588	ABF85681.1	31,73	4	4	1,13E+03	1	1	1	Dehydration	26007	cathepsin L3 partial [Fasciola hepatica]
19	2589	CAC12807.1	31,73	3	3	1,13E+03	1	1	1	Dehydration	35174	procathepsin L3 partial [Fasciola hepatica]
19	1253	ABW75768.2	31,73	3	3	1,13E+03	1	1	1	Dehydration	35718	procathepsin L partial [Fasciola hepatica]
19	123	ABZ80399.1	31,73	3	3	1,13E+03	1	1	1	Dehydration	35333	cathepsin L3 partial [Fasciola hepatica]
19	1254	ABW24657.1	31,73	3	3	1,13E+03	1	1	1	Dehydration	37418	cathepsin L [Fasciola hepatica]
19	1255	ACM67633.1	31,73	3	3	1,13E+03	1	1	1	Dehydration	37216	cathepsin 1L [Fasciola hepatica]
19	125	ACM67632.1	31,73	3	3	1,13E+03	1	1	1	Dehydration	37455	cathepsin 2L [Fasciola hepatica]
19	1256	QPX50259.1	31,73	3	3	1,13E+03	1	1	1	Dehydration	37306	cathepsin L3 peptidase [Fasciola hepatica]
22	182	THD19813.1	31,24	17	17	0	1	1	1	Carbamidome	8669	Cathepsin L [Fasciola hepatica]

FIGURE 7, LANE 4B

Protein Group	Protein ID	Accession	-10lgP	Coverage (%)	Coverage (%) 8	Area 8	#Peptides	#Unique	#Spec 8	PTM	Avg. Mass	Description
7	9	AAR99518.1	77,86	8	8	5,34E+03	3	1	3		36714	cathepsin L protein [Fasciola hepatica]
8	4	CAC12806.1	77,5	9	9	2,43E+03	3	1	3		35196	cathepsin L1 partial [Fasciola hepatica]
6	78	THD19412.1	44,24	9	9	1,56E+03	1	1	3		12490	Type-1 cystatin cysteine protease inhibitor [Fasciola hepatica]
6	79	AAV68752.1	44,24	9	9	1,56E+03	1	1	3		12472	cystatin [Fasciola hepatica]
6	77	pdb|6I1M|A	44,24	11	11	1,56E+03	1	1	3		9792	Chain A Cystatin
10	131	THD19897.1	42,99	10	10	0	1	1	2		11069	Stefin-1 [Fasciola hepatica]
11	200	THD27851.1	31,1	1	1	0	1	1	1		82596	T-cell immunomodulatory protein [Fasciola hepatica]

FIGURE 7, LANE 4C

Protein Group	Protein ID	Accession	-10lgP	Coverage (%)	Coverage (%) 9	Area 9	#Peptides	#Unique	#Spec 9	PTM	Avg. Mass	Description
2	9	AAR99518.1	100,11	11	11	5,21E+03	5	1	14		36714	cathepsin L protein [Fasciola hepatica]
3	4	CAC12806.1	98,54	12	12	2,22E+03	5	1	11		35196	cathepsin L1 partial [Fasciola hepatica]
8	78	THD19412.1	52,16	15	15	8,97E+02	2	2	2		12490	Type-1 cystatin cysteine protease inhibitor [Fasciola hepatica]
8	79	AAV68752.1	52,16	15	15	8,97E+02	2	2	2		12472	cystatin [Fasciola hepatica]
8	77	pdb|6I1M|A	52,16	18	18	8,97E+02	2	2	2		9792	Chain A Cystatin
5	13	pdb|2VYW|A	51,1	13	13	0	2	2	3		16560	Chain A HEMOGLOBIN
5	14	THD25867.1	51,1	13	13	0	2	2	3		16691	Myoglobin 2 [Fasciola hepatica]
5	15	ABW96608.1	51,1	13	13	0	2	2	3		16691	hemoglobin F2 [Fasciola hepatica]
5	12	THD25868.1	51,1	13	13	0	2	2	3		16722	Myoglobin 2 [Fasciola hepatica]
7	131	THD19897.1	44,96	10	10	0	1	1	2		11069	Stefin-1 [Fasciola hepatica]
15	206	THD29031.1	29,81	1	1	0	1	1	1		94546	Basement membrane-specific heparan sulfate proteoglycan core protein [Fasciola hepatica]
16	175	AAB46830.1	26,49	12	12	0	1	1	1		6589	Fh-KTM=6.751 kda monomeric Kunitz-type proteinase inhibitor [Fasciola hepatica=trematode Peptide 58 aa
16	176	THD28325.1	26,49	8	8	0	1	1	1		9314	Kunitz-CH [Fasciola hepatica]
16	177	CEL12048.1	26,49	8	8	0	1	1	1		9252	Kunitz [Fasciola hepatica]
16	178	CEL12049.1	26,49	8	8	0	1	1	1		9356	Kunitz-CH [Fasciola hepatica]
17	9930	THD18018.1	26,25	2	2	0	1	1	1		54766	Cyclin dependent kinase 17 partial [Fasciola hepatica]
17	9931	THD18040.1	26,25	2	2	0	1	1	1		54766	Cyclin dependent kinase 17 partial [Fasciola hepatica]
10	1255	ACM67633.1	25,48	3	3	0	1	1	1	Dehydration	37216	cathepsin 1L [Fasciola hepatica]
10	1256	QPX50259.1	25,48	3	3	0	1	1	1	Dehydration	37306	cathepsin L3 peptidase [Fasciola hepatica]
10	2588	ABF85681.1	25,48	4	4	0	1	1	1	Dehydration	26007	cathepsin L3 partial [Fasciola hepatica]
10	197	ABZ80402.1	25,48	3	3	0	1	1	1	Dehydration	34581	cathepsin L6 partial [Fasciola hepatica]
10	2589	CAC12807.1	25,48	3	3	0	1	1	1	Dehydration	35174	procathepsin L3 partial [Fasciola hepatica]
10	1253	ABW75768.2	25,48	3	3	0	1	1	1	Dehydration	35718	procathepsin L partial [Fasciola hepatica]
10	1254	ABW24657.1	25,48	3	3	0	1	1	1	Dehydration	37418	cathepsin L [Fasciola hepatica]
10	123	ABZ80399.1	25,48	3	3	0	1	1	1	Dehydration	35333	cathepsin L3 partial [Fasciola hepatica]
10	125	ACM67632.1	25,48	3	3	0	1	1	1	Dehydration	37455	cathepsin 2L [Fasciola hepatica]
11	568	THD21424.1	24,99	1	1	1,56E+03	1	1	1		79063	putative transcription factor LCR-F1 [Fasciola hepatica]
12	557	THD29090.1	24,93	1	1	0	1	1	1		59690	SoxB protein [Fasciola hepatica]
9	200	THD27851.1	24,65	1	1	5,09E+03	1	1	1		82596	T-cell immunomodulatory protein [Fasciola hepatica]
713	20876	THD20876.1	24,23	4	4	0	1	1	1	Carbamidomethylation; Oxidation (M); Dehydration	78543	hypothetical protein D915_008256 [Fasciola hepatica]

Figure S1:

Analysis by SDS-PAGE of the proteins contained in the HAC-NR, Peak IV and Fi-sole fractions (in triplicate). The photograph on the left shows a full view of the gel before the bands were cut for MS/MS analysis. The edges of the gel were framed within a black rectangle. The regions framed in red were used to compose the Figure 7 of the manuscript. The figure on the right shows the gel after cutting the bands of interest.

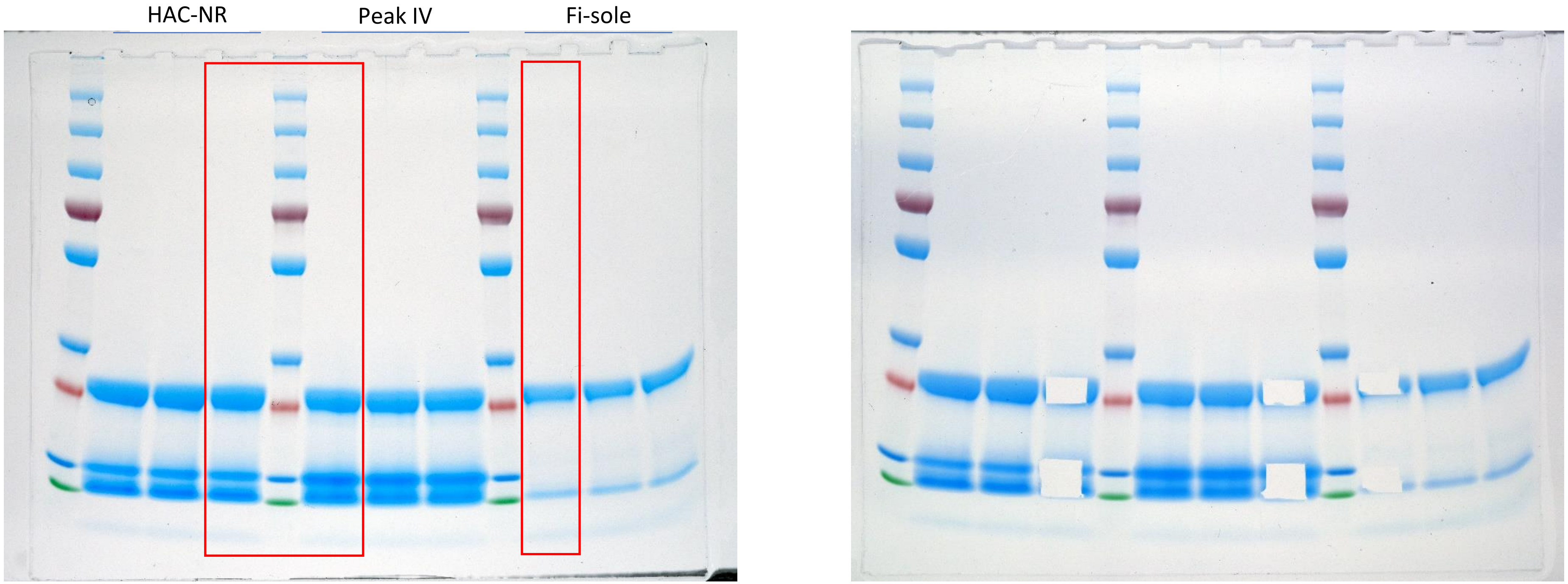

Supplement: Supplementary file 1 — Supplementary Information. [file 41598_2024_54290_MOESM1_ESM.pdf]
